# Linear Interaction Energy Based Prediction of Cytochrome P450 1A2 Binding Affinities with Reliability Estimation

**DOI:** 10.1371/journal.pone.0142232

**Published:** 2015-11-09

**Authors:** Luigi Capoferri, Marlies C. A. Verkade-Vreeker, Danny Buitenhuis, Jan N. M. Commandeur, Manuel Pastor, Nico P. E. Vermeulen, Daan P. Geerke

**Affiliations:** 1 AIMMS Division of Molecular Toxicology, Department of Chemistry and Pharmaceutical Sciences, VU University, De Boelelaan 1083, 1081 HV Amsterdam, The Netherlands; 2 Research Programme on Biomedical Informatics (GRIB), Department of Experimental and Health Sciences, Universitat Pompeu Fabra, IMIM (Hospital del Mar Medical Research Institute), Dr. Aiguader, 88, E-08003 Barcelona, Spain; Instituto de Tecnologica Química e Biológica, UNL, PORTUGAL

## Abstract

Prediction of human Cytochrome P450 (CYP) binding affinities of small ligands, i.e., substrates and inhibitors, represents an important task for predicting drug-drug interactions. A quantitative assessment of the ligand binding affinity towards different CYPs can provide an estimate of inhibitory activity or an indication of isoforms prone to interact with the substrate of inhibitors. However, the accuracy of global quantitative models for CYP substrate binding or inhibition based on traditional molecular descriptors can be limited, because of the lack of information on the structure and flexibility of the catalytic site of CYPs. Here we describe the application of a method that combines protein-ligand docking, Molecular Dynamics (MD) simulations and Linear Interaction Energy (LIE) theory, to allow for quantitative CYP affinity prediction. Using this combined approach, a LIE model for human CYP 1A2 was developed and evaluated, based on a structurally diverse dataset for which the estimated experimental uncertainty was 3.3 kJ mol^-1^. For the computed CYP 1A2 binding affinities, the model showed a root mean square error (RMSE) of 4.1 kJ mol^-1^ and a standard error in prediction (SDEP) in cross-validation of 4.3 kJ mol^-1^. A novel approach that includes information on both structural ligand description and protein-ligand interaction was developed for estimating the reliability of predictions, and was able to identify compounds from an external test set with a SDEP for the predicted affinities of 4.6 kJ mol^-1^ (corresponding to 0.8 p*K*_*i*_ units).

## Introduction

Cytochrome P450s (CYPs) form a ubiquitous superfamily of monooxygenases characterized by the presence of a heme cofactor, that in humans plays a crucial role in phase I drug metabolism [1]. Besides being responsible for about 50% of drug clearance via metabolism, CYPs can also be responsible for prodrug activation or metabolism-dependent toxicity [2]. Furthermore, their inactivation or inhibition can alter the metabolic pathway of co-administered drugs, potentially leading to drug-drug interactions (DDI). In the past few years this has been the cause of removal from the market of several drugs [1–3]. While *in vitro* screening for CYP binders and inhibitors is well established as a mean for predicting potential (adverse) drug-drug interactions *in vivo* [2,3], the interest for *in silico* methods has recently increased as a fast preliminary screening method in the drug discovery process [4]. However, these methods are still challenged by the substrate promiscuity and large catalytic site malleability of many CYP isoforms, including *e*.*g*. drug metabolizing CYP 3A4, 2D6 and 1A2 [4,5].

The broad substrate selectivity of CYPs is due to the large flexibility of their buried catalytic site, which allows many isoforms both to interact with different classes of molecules and to bind the same molecule in multiple orientations, thus enabling the formation of different metabolites of a single compound [6]. Variation in available experimental data is often an additional element of complexity in developing predictive models for binding of ligands (*i*.*e*. substrates and/or inhibitors) to CYPs. Several assays have been developed for screening inhibitory activity of small molecules toward CYPs, and use of different types of conditions (solvent composition, substrate probe, expression system, etc.) has been demonstrated to affect the outcome of the measurements [7,8]. Furthermore, CYP inhibitors can act via different mechanisms, including (1) competitive and reversible inhibition, (2) quasi-irreversible inhibition due to coordination with the heme iron, and (3) mechanism-based inhibition, in which an intermediate or product of the catalysis covalently modifies and inactivates the enzyme [9]. Experiments that allow to identify the inhibition mechanism of a compound are not performed routinely. Usually, relative *IC*_*50*_ values, measured under specific conditions, are reported in literature (instead of measuring absolute inhibition constants), and studies on the mechanism of inhibition are mostly omitted.

Despite of the major challenges in modeling CYP binding introduced above, several computational models have been proposed to model inhibition data categorically or quantitatively, in terms of *IC*_*50*_ values or inhibition constants (*K*_*i*_) [4].

In this regard, quantitative structure-activity relationship (QSAR) models based on molecular descriptors typically can show predictivity for small series of structurally related molecules, but are not suited for structurally diverse compounds. Using homogeneous experimental data for ~1500 diverse compounds for the most relevant CYP isoforms, Gleeson *et al*. [10] showed that the ability to describe the extent of CYP inhibition through traditional QSAR descriptors was limited and poor, since the role played by the molecular recognition process was neglected. Mathematical models including pharmacophore elements have been designed with higher predictivity. Similarly, 3D-QSAR and structure-based models that take into account direct information about the active site were also proposed, resulting in predictive *local* models; exhaustive reviews on QSAR models to predict inhibition of CYPs have been published [4,11].

Some years ago, efforts started in our laboratory to develop dynamical structural models for the prediction of the free energy of binding (Δ*G*_*bind*_) as a measure for ligand binding affinity and, therefore, the inhibitory potency, for series of small molecules toward specific CYP isoforms. In these models, molecular dynamics (MD) simulations are performed of the CYP-ligand complex in different representative binding conformations obtained from molecular docking. To obtain quantitative binding affinity prediction, we have applied an iterative version of Linear Interaction Energy (LIE) theory [12] as introduced by Stjernschantz and Oostenbrink [13]. The high level of detail offered by this approach clearly represents a trade-off in the accuracy of prediction versus computational time expended, for which accessibility has anyway increased in the last years. Moreover, by combining and (re)weighting results from multiple short simulations starting from different protein-ligand configurations, LIE predictions for flexible proteins such as CYPs become not only more accurate but also faster [13–15].

However, a general limitation to the extensive application of any structure-based model is the difficulty in estimating the reliability of a prediction, which can be easier provided for pure statistical models. In the case of ligand-based QSAR models, for instance, a prediction is considered reliable when the query compound falls within the area of multidimensional space defined by the molecular descriptors employed by the model that has already been explored by the training set. This evaluation can be carried out in different ways, according to the method applied to interpolate the space defined by the descriptors [16]. In this context, Carriò *et al*. [17] recently proposed an approach (ADAN) in which several similarity criteria are taken into account to analyze this space, and a query compound can be classified in a reliability category according to the number of the criteria for which the compound is considered outlier. In the current work we introduce an approach to enable reliability estimation of LIE predictions, by using criteria that involve not only the ligand description but also a comparison between protein-ligand interactions sampled by the training and query compounds during MD.

In humans, isoform CYP 1A2 represents approximately 15% of the total CYP liver content and is responsible for 5% of CYP metabolism of the currently marketed drugs [2]. Furthermore, it is responsible for metabolism of many other exogenous and endogenous compounds, such as caffeine, teophylline, steroids, aromatic and heterocyclic amines, and polycyclic aromatic hydrocarbons (PAHs). Inhibitors of CYP 1A2 are usually planar and lipophilic, and are often small-volume molecules that are neutral or weakly basic with few hydrogen bonds donors [18,19]. Consistently, the X-ray resolved structure of this enzyme [20] showed a narrow active site in which residues of helix F and helix I form two parallel substrate binding platforms that are able to allocate flat lipophilic molecules. Additionally, Thr_118_, Ser_122_, Thr_124_ and Asp_321_ on one side of the active site, and Thr_223_ and Asp_320_ on the other, define two areas in which ligands can undertake polar interactions.

Previously reported statistical models based on molecular descriptors gave good performance in qualitative prediction of CYP 1A2 inhibition [21,22], or for quantitative estimation of the inhibitory potency for local classes of compounds (flavonoids and polycyclic aromatic compounds) [23,24]. CoMFA and GRID/GOLPE models have also been developed to enable estimation of inhibitory potency estimation for sets of chemically related compounds [25,26].

In a previous study, a quantitative model for CYP 1A2 ligand-binding affinity prediction was obtained using LIE and a training set of 8 *structurally diverse* compounds [27]. While the choice of the initial pose in setting up the MD simulations and LIE calculations was knowledge based, the result of the investigation indicated that such technique could be successfully applied in the development of global quantitative models for prediction of CYP 1A2 binding affinities and hence, inhibition.

Here we present a comprehensive quantitative model for the prediction of the affinity (free energy) of (reversible) binding of drug-like compounds toward CYP 1A2. Our model is based on the iterative LIE method [13–15] and does not require any *a priori* knowledge other than the CYP 1A2 crystal structure [20] and *IC*_*50*_ (or *K*_*i*_) data for calibration. For this purpose, a database of (> 50) molecules was collected from literature and for several compounds from each source, we experimentally determined CYP 1A2 inhibition in our laboratory to evaluate the experimental uncertainty among the different sources used. The dataset was split in a training and test set, and a LIE affinity model was calibrated. Finally, we used our criteria driven approach that accounts for both the nature of the ligands and the protein-ligand interactions, in order to evaluate the quality of external LIE predictions. For that purpose, a set of analyses was performed that was considered representative for the high number of factors playing a role in the computation of LIE predictions. The results obtained suggest that this novel approach, here applied to the CYP 1A2 affinity model, can also represent a general method for estimating the reliability of predictions from other LIE models.

## Methods

### Chemicals

Mefenamic acid (≥99%), tacrine (≥99%), carvedilol (≥98%), nifedipine (≥98%), ellipticine, α-naphthoflavone (≥97%), ticlopidine (≥99%), 1-naphtol (≥99%), 2-naphtol (≥98%), 4-methoxy-benzaldehyde (≥98%), 2-(p-tolyl)ethylamine (≥97%) were purchased from Sigma-Aldrich (Schnelldorf, Germany); phenacetin was obtained from Brocades-ACF (Maarssen, the Netherlands). 7-Methoxyresorufin was synthesized by the method of Burke and Mayer [28] and final purity was assessed to be higher than 95%.

### Enzyme expression

The plasmid containing the human CYP 1A2 cDNA and human NADPH CYP reductase was transformed into *Escherichia coli* strain DH5α. CYPs were expressed in 3-L flasks containing 300 mL terrific broth (TB) with 1 mM δ-aminolevulinic acid, 0.5 mM thiamine, 400 μL/L trace elements, 100 μg/mL ampicillin, 1 mM isopropyl-β-d-thiogalactopyranoside (IPTG), and 0.5 mM FeCl_3_. The culture media was inoculated with 3 mL overnight culture. The cells were allowed to grow for 40 h at 28°C and 125 rpm. *E*. *coli*’s were collected by centrifugation (4000 × *g*, 4°C, 15 min) and resuspended in 20 mL 0.1 M potassium phosphate (KPi) glycerol buffer at pH 7.4 (containing 20% glycerol v/v, 0.25 mM ethylenediaminetetraacetic acid (EDTA), and 0.1 mM dithiothreitol (DTT)). Prior to cell disruption by emulsiflex (3 repeats), cells were treated with 0.5 mg/mL lysozyme. The membranes containing the human CYP were isolated by ultracentrifugation in a Beckmann 70Ti rotor (75 min, 40,000 rpm, 4°C), resuspended in KPi-glycerol buffer, and subsequently homogenized by pottering. The CYP 1A2 concentration was determined using the method of Omura and Sato [29] and the enzyme was stored at -80°C until use.

### Inhibition assay

The inhibitory activity of several compounds towards human CYP 1A2 was determined using 7-methoxyresorufin as a substrate. 7-Methoxyresorufin was used at its *K*_*m*_ value, which was determined to be 2.5 μM (data not shown). Incubations were carried out in a total volume of 200 μL and in the presence of an NADPH regenerating system (NRS) (final concentrations of 0.5 mM NADPH, 10 mM glucose 6-phosphate, and 0.4 unit/mL glucose-6-phosphate dehydrogenase) in a black coaster 96-well plate. CYP 1A2 was pre-incubated for 5 minutes at 37°C with 0.1 M potassium phosphate buffer (pH 7.4), 7-methoxyresorufin and inhibitors, with DMSO at a final concentration of 0.5% (v/v). For the *IC*_*50*_ determinations the inhibitor concentration was varied between 10 pM and 10 mM, and NRS was added to start the reaction. Resorufin formation was followed fluorimetrically in time for 10 minutes on a Victor2 1420 multilabel counter with excitation at 530 nm and emission at 572 nm. A resorufin calibration curve was used to quantify the amount of product formed. All measurements were performed in triplicate.

### Set-up of MD simulations and development of a CYP 1A2 LIE model

Using (iterative) LIE, affinity prediction requires preparation and selection of ligand poses, setting up and running MD simulations, and model calibration and binding free energy calculations [15]. Computational settings and methodological details as applied in the current work are given below, together with details for the criteria used to evaluate the predictive quality of (iterative) LIE models.

### Ligand preparation

For all training and test set compounds, inhibition constants *K*_*i*_ were derived from *IC*_*50*_ values from literature according to the Cheng-Prusoff equation [30], and experimental values for the free energy of binding Δ*G*_*bind*_ were calculated as

$$\Delta G_{bind}=RT\;\text{ln}\left(K_{i}\right).$$ (1)

The initial structure of each ligand was generated using Molecular Operating Environment (MOE) 2012.10 [31] and subsequently neutralized and minimized in OpenBabel 2.3.2 [32] using the MMFF94 force field [33]. The geometry was further optimized by the sqm module of Ambertools [34] at the AM1-BCC [35] semi-empirical level of theory. Molecular structures and literature *IC*_50_ values are reported in Table A in S1 File.

### Docking and clustering

The crystal structure of CYP 1A2 was obtained from the Protein Data Bank (code: 2HI4) [20] and refined as described in reference 27. Each ligand was docked into the active site of CYP 1A2 using PLANTS (Protein-Ligand Ant System) version 1.2 [36], and the ChemPLP [37] scoring function. The center of the docking sphere was placed at a distance of 0.78 nm from Fe, along the vector connecting the sulphur atom of the Fe-coordinated cysteine (Cys_458_) and the heme iron, from which the radius was set to 1.2 nm to define the active site of the protein. Maximally 300 docked poses with mutual root-mean-square deviations (RMSDs) in atomic positions of > 0.2 nm were retained.

Coordinates of the heavy atoms were used as variables for a principal component analysis (PCA) of the docked poses. After dimensionality reduction, the scores obtained were used for subsequent *k-means* clustering [38]. During this analysis, an additional component or cluster was taken into account in case it would have led to a further increment of at least 5% of the explained variance in the space of the coordinates or scores, respectively. The medoids of the clusters obtained (4 to 7 per ligand) were chosen as representative binding-conformations of the ligand in the CYP 1A2 active site. These configurations were used to filter out potential non-competitive inhibitors and as starting poses for the MD simulations used in the LIE model, as described below.

### Identification of (quasi-)irreversible binders

Mechanism-based inhibitors and heme coordinating agents are characterized by specific chemical groups (Table B in S1 File) that can lead to (quasi-)irreversible inhibition in case they can interact with or be activated by the heme iron [9]. The presence of these groups in the ligands was evaluated and in case the corresponding reactive atom center was found closer than 0.6 nm from the heme iron in at least one of the representative poses, a ligand was considered a quasi-irreversible or mechanism-based inhibitor and it was excluded from the dataset.

### MD simulations

Every representative binding pose obtained from clustering was used as starting configuration for use in MD. Simulations were carried out using the GROMACS 4.5.5 package [39]. Topologies of the ligands were automatically created by ACPYPE [40] using the General Amber Force Field [41] as potential, while the Amber99SB [42] force field was applied to describe the protein. To model the heme group, force field parameters reported in reference [43] were used. Each complex was solvated in a dodecahedral box filled with TIP3P water [44] (~20,000 solvent molecules), and 7 Cl^-^ ions were added to neutralize the system. The system was energy minimized and gradually heated up to 300 K in three *NVT* simulations of 20 ps at 100K, 200K, and 300 K, respectively, in which harmonic potentials were used to positionally restrain C_α_ atoms (with force constants of 10000, 5000, and 50 kJ nm^-2^ mol^-1^, respectively) and other heavy atoms (with force constants of 2000, 1000, and 10 kJ nm^-2^ mol^-1^, respectively). Subsequently, 2.5 ns of unrestrained *NpT* simulations were performed at 1.01325 bar and 300 K, of which the first 0.5 ns were discarded in further analyses. A leap-frog algorithm was employed for integrating the equations of motion [45]. Heavy hydrogens (with a mass of 4.032 amu) [46] were used and all bonds were constrained using the LINCS algorithm [47], allowing a time-step of 4 fs. A Berendsen thermostat [48] was employed to maintain the temperature of the system close to its reference value, using separate temperature baths for the solvent and solute degrees of freedom, with a coupling time of 0.1 ps. A Berendsen barostat [48] with a coupling time of 0.5 ps and an isothermal compressibility of 4.5×10^−5^ bar^-1^ was used to maintain the pressure close to its reference value during *NpT* simulations. Van der Waals and short-range electrostatic interactions were explicitly evaluated every time step for pairs of atoms within a 0.9 nm cutoff, and a grid-based neighbor list was used and updated every 2 time steps. Long-range electrostatic interactions were included by using the smooth particle mesh Ewald method [49] with a maximum fast Fourier transform grid spacing of 0.125 nm for the reciprocal space sum.

To evaluate average ligand interaction energies of the unbound ligands in water, each ligand was solvated in a dodecahedral box filled with approximately 650 TIP3P water molecules, and no counterions were added. The MD protocol was identical to the one described for the simulations of the protein-ligand complex.

### LIE model

According to LIE theory [12], Δ*G*_*bind*_ can be calculated from differences (Δ*V* ^*Ele*^ and Δ*V* ^*VdW*^) in the ensemble-averaged electrostatic ⟨*V* ^*Ele*^_*lig−surr*_⟩ and van der Waals interaction energies ⟨*V* ^*VdW*^_*lig−surr*_⟩ between the ligand and its surroundings when simulated in complex with the protein, or in the free state (water). Δ*G*_*bind*_ of the ligand to the protein can be calculated as [12]

$$\begin{array}{l} \Delta G_{bind}=\alpha \left(\langle V_{lig-surr}^{VdW}\rangle {}_{protein}-\langle V_{lig-surr}^{VdW}\rangle {}_{water}\right)+\beta \left(\langle V_{lig-surr}^{Ele}\rangle {}_{protein}-\langle V_{lig-surr}^{Ele}\rangle {}_{water}\right)\\ \;=\alpha \;\Delta V^{VdW}+\beta \;\Delta V^{Ele} \end{array}$$ (2)

In Eq 2, *α* and *β* are empirical parameters for the van der Waals and electrostatic contribution of the nonbonded interactions to Δ*G*_*bind*_. An offset parameter (*γ*) might also be considered, although not strictly required for calculation of relative binding affinities. The value of such parameter has been related to the hydrophobicity of the binding site [50], and in any case it has to be determined empirically as well.

When combining results from several MD simulations per ligand that start from different protein-ligand configurations, a relative contribution *W*_*i*_ of each simulation *i* to the total calculated interaction energies of the ligand can be determined as [13,51]

$$W_{i}=\frac{e^{-\Delta G_{i}/k_{B}T}}{\sum_{i}\;e^{-\Delta G_{i}/k_{B}T}}\;,$$ (3)

where Δ*G*_*i*_ is the Δ*G*_*bind*_ value for simulation *i*, obtained from Eq 2. Using the *W*_*i*_’s, Δ*G*_*bind*_ of the ligand averaged over the independent simulations, *i* can be calculated as

$$\Delta G_{bind}\;=\;\alpha \;\sum_{i}\;W_{i}\Delta V_{i}^{VdW}+\beta \;\sum_{i}\;W_{i}\Delta V_{i}^{Ele}\;.$$ (4)

During model training, the *W*_*i*_’s are obtained by applying an iterative scheme, as described by Stjernschantz and Oostenbrink [13].

### Chemical similarity analysis

For each compound, a molecular fingerprint was created according to the MACCS smart pattern [52]. Similarity among the compounds was evaluated in terms of Tanimoto Scores (TSs) between pairs of fingerprints [53] using RDKit release_2014.09.1 [54]. Every training compound was compared with the other elements of the training set and the TS with the most similar compound was stored for every ligand. The lowest value was used as cut-off for the test compounds when compared to the most similar molecule of the training set.

### Nonbonded energy terms distribution analysis

The set of simulations employed in the LIE model were analyzed in terms of Δ*V* ^*Ele*^ and Δ*V* ^*VdW*^, as described in reference 15. For each simulation of the test set compounds, the Mahalanobis distance was computed from the center of the distribution of Δ*V* ^*Ele*^ and Δ*V* ^*VdW*^ values obtained for the simulations used to train the LIE model. Supposing a uniform distribution of the energies for the training set, a query compound was considered outlier within this analysis if at least one simulation showed a squared distance higher than those expected for the 95 percentile of the *χ* ^2^ distribution for one degree of freedom (*i*.*e*., Δ*V* ^*Ele*^ or Δ*V* ^*VdW*^) [55].

### Per-residue interaction energy decomposition

Nonbonded interaction energies of the ligand with specific residues surrounding the catalytic site were analyzed during the simulations of the protein-ligand complexes. Residues included in the analysis were selected on the basis of the distance of any of their atoms to the center of the catalytic site. The center of the catalytic site was defined as the center for docking and the cut-off radius to select relevant residues was 1.2 nm. Ensemble nonbonded energy contributes for each residue over the MD simulations were scaled per simulation by the weights *W*_*i*_ obtained from the reweighting scheme described above and summed, giving a single set of contributions per residue for each ligand.

PCA was performed for the elements of the training set in the space of the residue-decomposed energy contributes, after preliminary centering of the variables. Components that explained more than 5% of the variance in the original space were taken into account. Elements of the test set were projected on the rotated space and evaluated for being score or orthogonal outliers. The critical score distance (*SD*_*crit*_) was calculated as square root of the 95 percentile of the *χ* ^2^ distribution for *a* degrees of freedom, corresponding to the number of principal components:

$$SD_{crit}=\sqrt{\chi_{a,0.95}^{2}}$$ (5)

The orthogonal critical distance (*OD*_*crit*_) was robustly measured as

$$OD_{crit}={\left(median{\left(OD\right)}^{2/3}+MAD\left(OD^{2/3}\right)\cdot z_{0.95}\right)}^{3/2}$$ (6)

where *MAD* is the median absolute deviation and *z* the 95 percentile of the cumulative normal distribution [56].

## Results and Discussion

### Assessing the experimental uncertainty

Inhibition studies in literature typically present data for relatively small sets of structurally related compounds. To calibrate a model for the prediction of binding affinity for different classes of molecules, we gathered experimental data from three different literature sources, providing a dataset of 73 compounds. Source 1 presents *IC*_*50*_ data for 14 marketed drugs that have been screened internally by *Bayer HealthCare*, according to FDA and UP guidelines, using phenacetin as substrate probe on pooled human liver microsomes [57]. Source 2 consists of eight drugs and common inhibitor probes of CYP 1A2, screened for the inhibitory activity against phenacetin O-deethylation on recombinant CYPs [58]. In Source 3, 51 lactones or substituted naphthalenes and quinolines were screened for their inhibitory potency in a 7-ethoxyresorufin O-dealkylation assay on recombinant CYP 1A2 [25,59]. A sample of four compounds from each dataset was screened in-house, in order to estimate the expected error for a computational model based on the experimental uncertainty. The results from this comparison between our *IC*_*50*_ data and data from Sources 1–3 are presented in Table 1, and show that only for compounds 1 and 6–9 the difference in the values from the different assays was within the experimental error. Representing the affinity as Δ*G*_*bind*_, the root mean square deviation (RMSD) in experimental affinity is 3.3 kJ mol^-1^ over the full set of measurements, with deviations spanning between 0.4 and 6.9 kJ mol^-1^.

**Table 1:** Comparison of *IC*_*50*_ and Δ*G*_*bind*_ values for CYP 1A2 as determined in-house (inhouse) and gathered from literature sources (lit), expressed in μM and kJ mol^-1^, respectively. **ΔΔ*G***_***bind***_ **refers to the difference in Δ*G***_***bind***_ **between literature and in-house determined values.**

ID	Compound	*IC*_*50*_ (inhouse)	*IC*_*50*_ (lit)	Δ*G*_*bind*_ (inhouse)	Δ*G*_*bind*_ (lit)	ΔΔ*G*_*bind*_
1	Mefenamic acid^a^	19 ± 7	13.98	-28.9 ± 1.0	-29.7	-0.8
2	Tacrine^a^	2.3 ± 1.0	5.2	-34.2 ± 1.2	-32.1	2.1
3	Carvedilol^a^	5.0 ± 0.2	5.91	-32.2 ± 0.1	-31.8	0.4
4	Nifedipine^a^	0.7 ± 0.2	5.74	-37.2 ± 0.7	-31.9	5.3
5	Ellipticine^b^	0.007 ± 0.002	0.11 ± 0.01	-48.7 ± 0.7	-41.8 ± 0.2	6.9
6	Phenacetin^b^	24 ± 6	28.0 ± 0.46	-28.3 ± 0.6	-27.9 ± 0.0	0.4
7	α-Naphthoflavone^b^	0.030 ± 0.026	0.08 ± 0.02	-45.0 ± 3.7	-42.6 ± 0.6	2.4
8	Ticlopidine^b^	12 ± 7	6.1 ± 0.45	-30.1 ± 1.7	-31.8 ± 0.2	-1.7
9	1-naphthol^c^	2.0 ± 0.7	3.2 ± 0.8	-34.5 ± 0.9	-33.3 ± 0.6	1.2
10	2-naphthol^c^	4.4 ± 1.2	17 ± 1.5	-32.6 ± 0.7	-29.1 ± 0.2	3.4
11	4-methoxy-benzaldehyde^c^	410 ± 32	270 ± 85	-21.2 ± 0.2	-22.2 ± 0.8	-1.0
12	2-(p-tolyl)ethylamine^c^	120 ± 33	14 ± 2.5	-24.3 ± 0.7	-29.6 ± 0.5	-5.3

^a^Literature data from Ref. 57;

^b^Literature data from Ref. 58;

^c^Literature data from Ref. 25.

The analysis presented here provides an estimate of the variability in measured *IC*_*50*_ values under the different experimental conditions adopted in the studies from which CYP 1A2 inhibition potencies have been gathered. Based on the fact that the uncertainty of any quantitative model is dependent on the uncertainty of the experimental data on which it is based, the observed experimental variability is used as a parameter to evaluate the quality of our model. According to this, a non-overfitted reliable model is expected to show root mean regression errors (RMSE) of at least 3.3 kJ mol^-1^ and maximum errors in regression of 6.9 kJ mol^-1^ (corresponding to an error in p*K*_*i*_ of approximatively 0.6 and 1.2, respectively, Eq 1).

### Restriction of the dataset to competitive binders

Compounds from the data set were docked into the CYP 1A2 catalytic site and clustered in order to obtain representative binding poses for each compound.

It is known that several classes of CYP inhibitors can act in a (quasi-)irreversible manner: aromatic nitrogens can coordinate the heme iron, while biotransformation by CYPs can lead to reactive intermediates and subsequent covalent modification and inactivation of the enzyme itself. In these cases, quantum-mechanic (QM) based corrections should be used to address all the energetic contributes involved in bond breaking and formation. To exclude compounds that would not be properly described by the molecular mechanics (MM) force fields used in MD and LIE, a preliminary filter combining structural information from docking and knowledge-based rules was developed.

The binding poses obtained from docking and clustering were inspected and compounds were excluded from further analysis when bearing chemical groups that are known to lead to quasi-irreversible or mechanism-based inhibition (Table B in S1 File), *and* when (any of) the reactive atom(s) was within 0.6 nm from the heme iron.

Literature data [60–73] on the mechanism of inhibition are available for 14 compounds of the dataset, Table 2. In case of discordant literature data, information from the most recent reference was taken into account. Compounds that were known from literature to be a substrate were here considered as competitive inhibitors, since they can potentially compete for binding to the CYP with other substrates. Our preliminary filter was able to correctly classify 10 of the 14 inhibitors considered (Table 2), while carvedilol, ticlopidine, clopidogrel, and riluzole were wrongly classified as mechanism-based inhibitors. Both clopidogrel and riluzole are metabolized by CYP 1A2 at a chemical position that is considered to lead to mechanism-based inhibition [69,73] representing exceptions to the general rule. Furthermore, our approach showed high precision in detecting competitive inhibitors, since no false positives were detected (Table 2).

**Table 2:** Comparison between predicted inhibition mechanisms and experimentally determined inhibition mechanisms reported in literature.

ID	Compound	Predicted	Reported
1	Mefenamic acid	Competitive	Competitive^60^
2	Tacrine	Competitive	Substrate^61^
3	Carvedilol	Quasi-irreversible	Substrate^62^
4	Nifedipine	Competitive	Competitive^63^
6	Phenacetin	Competitive	Substrate^64^
7	α-Naphthoflavone	Competitive	Competitive/uncompetitive^65^
8	Ticlopidine	Mechanism-based	Competitive^66^
14	Quercetin	Competitive	Competitive^67^
17	Naringenin	Competitive	Substrate^68^
19	Clopidogrel	Mechanism-based	Substrate^69^
23	Mexiletine	Competitive	Competitive^70^
24	Furafylline	Mechanism-based	Mechanism-based^71^
25	Propranolol	Competitive	Substrate^72^
26	Riluzole	Mechanism-based	Substrate^73^

While an extensive validation of our filtering approach is beyond the scope of this work, it showed to be accurate enough to be employed as a preliminary filter for building a LIE-based model. After refinement of the entire dataset, 57 compounds were predicted to be competitive inhibitors of CYP 1A2 and subsequently simulated by MD in solvent and in complex with the enzyme, starting from every of the representative binding modes obtained from docking (Table C in S1 File).

### The LIE model

**Fig 1:**
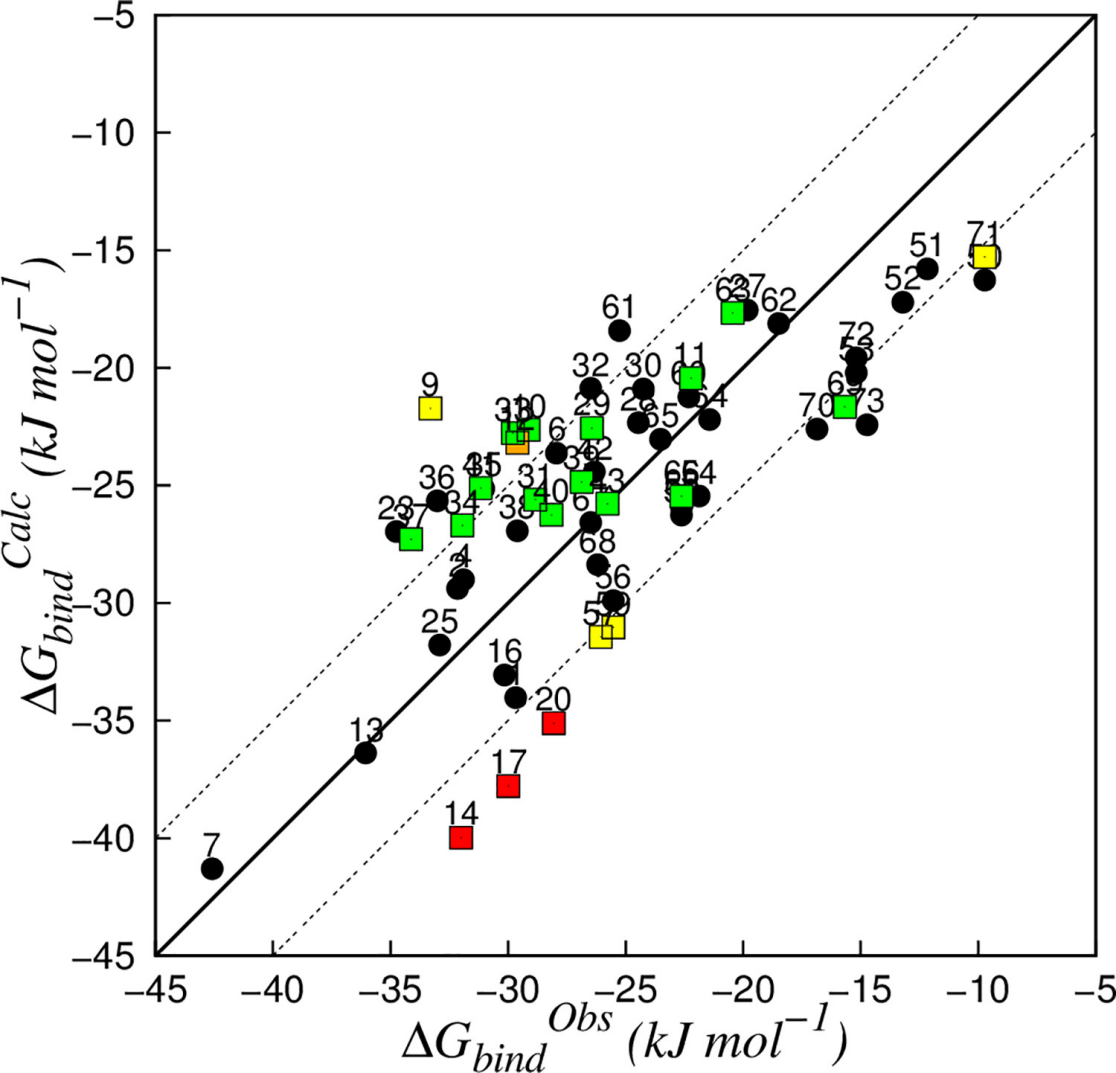
Correlation between calculated (∆*G_bind_* ^*Cal*c^) and observed (∆*G_bind_^Obs^*) binding free energies obtained for the CYP 1A2 LIE model (Eq 4, *α* = 0.587 and *β* = 0.267). The solid line indicates ideal correlation between ∆*G_bind_^Obs^* and ∆*G_bind_^Calc^*, and dashed lines represent deviations between calculated and experimental values of ±5 kJ mol^−1^ (corresponding to an error well within 1.0 p*K*_*i*_ units). Compounds from the training set are represented in black. Test-set compounds that were found to be outlier in 0, 1, 2, and 3 analyses are represented in green, yellow, orange, and red, respectively.

Compounds of the refined dataset of 57 compounds were split in a training and test set. In order to ensure a broad chemical diversity within both data sets and a homogeneous representation of the different chemical classes, the compounds were classified according to their structure in naphthalenes/quinolines (compounds 9–10, 27–43), lactones (compounds 50–59) and other small molecules (others). From the three classes 8, 8, and 19 compounds were randomly selected to train the model (Table 3), respectively, while the other 22 compounds were used as external test set. An increasing number of MD simulations per ligand was taken into account to train several LIE models, using for each of them results from the simulations with the lowest Δ*G*_*bind*_. Among these models, the model with lowest standard error in prediction (SDEP) obtained during leave-one-out cross-validation (LOO-CV) was based on maximally 6 simulations per compound, and showed values of 0.587 and 0.267 for *α* and *β* in Eq 4, respectively (Fig 1). The RMSE and the SDEP for LOO-CV (SDEP_CV_) were 4.1 and 4.3 kJ mol^-1^, respectively, and were close to our estimate for the uncertainty in the experimental data (3.3 kJ mol^-1^). The 22 compounds that were not included in the training set were used to evaluate the predictive quality of the LIE model. The SDEP obtained for the 22 molecules of the test set was 5.8 kJ mol^-1^, with differences from experiments between 0.0 and 11.6 kJ mol^-1^ (Fig 1 and Table 4).

**Table 3:** Calculated (Δ*G_bind_^Calc^*) and observed (Δ*G_bind_^Obs^*) free energies of binding, and corresponding residuals (Δ*G_bind_^Obs^*—Δ*G_bind_^Calc^*) for the training-set compounds (kJ mol^-1^).

ID	Compound	Δ*G_bind_^Obs^*	Δ*G_bind_^Calc^*	Residual
1	Mefenamic Acid	-29.7	-34.0	4.3
2	Tacrine	-32.2	-29.4	-2.8
4	Nifedipine	-31.9	-29.0	-2.9
6	Phenacetin	-27.9	-23.6	-4.3
7	α-naphthoflavon	-42.6	-41.3	-1.3
13	HET-0016	-36.1	-36.4	0.3
16	Niflumic acid	-30.2	-33.1	2.9
23	Mexiletine	-34.8	-27.0	-7.8
25	Propranolol	-32.9	-31.8	-1.1
27	Naphthalene	-19.8	-17.6	-2.3
28	1-Methylnaphthalene	-24.5	-22.3	-2.1
30	2-Methylnaphthalene	-24.2	-20.9	-3.3
32	2-Fluoronaphthalene	-26.5	-20.9	-5.6
35	1,3-Dimethylnaphthalene	-31.0	-25.1	-5.9
36	1,4-Dimethylnaphthalene	-33.0	-25.7	-7.4
38	1,5Dichloronaphthalene	-29.6	-26.9	-2.7
42	2,6-Dimethylnaphthalen	-26.3	-24.4	-1.9
50	ε-Caprolactone	-9.7	-16.3	6.6
51	γ-Valerolactone	-12.2	-15.8	3.6
52	γ-Caprolactone	-13.2	-17.2	4.0
53	γ-Heptalactone	-15.2	-20.2	5.0
54	γ-Nonanoic-lactone	-21.9	-25.4	3.6
55	γ-Decanolactone	-22.6	-25.9	3.3
56	γ-Undecanolactone	-25.5	-29.9	4.4
58	δ-decanolactone	-22.6	-26.3	3.6
60	2-Coumarone	-22.3	-21.2	-1.1
61	2-Indanone	-25.3	-18.4	-6.8
62	2,3-Dihydrobenzofuran	-18.5	-18.1	-0.4
64	2-Benzoxalinone	-21.4	-22.2	0.8
65	Biphenyl	-23.5	-23.0	-0.5
67	4-Chlorobiphenyl	-26.5	-26.6	0.1
68	Butylcyclohexane	-26.2	-28.4	2.2
70	γ-Phenyl-γ-butyrolactone	-16.9	-22.6	5.7
72	2-(p-tolyl)ethylamine	-15.2	-19.6	4.4
73	Cotinine	-14.7	-22.4	7.7

**Table 4:** Calculated (Δ*G*_bind_^Calc^) and observed (Δ*G_bind_^Obs^*) free energies of binding (kJ mol^-1^), and residuals (Δ*G_bind_^Obs^*–Δ*G*_bind_^Calc^) for the test-set compounds. Results from the reliability analyses are given as well, where a score 1 in columns (A)-(D) refers to the identification of outliers according to the following analyses: (A) Chemical similarity analysis; (B) Average interaction energy distribution analysis; (C) Ligand-residue electrostatic interaction analysis; (D) Ligand-residue van der Waals interaction analysis. In the last column (Total), the total sum of the number of analyses is reported in which a compound is identified as an outlier.

				Outlier identification				
ID	Δ*G*_bind_^Obs^	Δ*G*_bind_^Calc^	Residual	A	B	C	D	Total
9	-33.3	-21.7	-11.6	0	0	1	0	1
10	-29.1	-22.6	-6.5	0	0	0	0	0
11	-22.2	-20.4	-1.8	0	0	0	0	0
12	-29.6	-23.2	-6.4	1	0	1	0	2
14	-32.0	-40.0	8.0	0	1	1	1	3
17	-30.0	-37.8	7.8	0	1	1	1	3
20	-28.1	-35.1	7.0	0	1	1	1	3
29	-26.4	-22.6	-3.9	0	0	0	0	0
31	-28.9	-25.6	-3.3	0	0	0	0	0
33	-29.8	-22.8	-7.0	0	0	0	0	0
34	-32.0	-26.7	-5.3	0	0	0	0	0
37	-34.1	-27.3	-6.8	0	0	0	0	0
39	-26.9	-24.9	-2.0	0	0	0	0	0
40	-28.2	-26.3	-1.9	0	0	0	0	0
41	-31.2	-25.1	-6.1	0	0	0	0	0
43	-25.8	-25.8	0.0	0	0	0	0	0
57	-26.1	-31.4	5.4	0	0	0	1	1
59	-25.5	-31.0	5.5	0	0	0	1	1
63	-20.4	-17.7	-2.8	0	0	0	0	0
66	-22.6	-25.5	2.8	0	0	0	0	0
69	-15.7	-21.7	6.0	0	0	0	0	0
71	-9.7	-15.3	5.6	1	0	0	0	1

Analysis of the relative weight *W*_*i*_ for each protein-ligand binding pose (Eq 3, Table C in S1 File) shows that most ligands present multiple binding conformations that significantly contribute to the calculated binding free energy. It is interesting to note that for this dataset of compounds and when not considering the physical meaning of including the contribution from more than one ligand-protein binding conformation to ∆*G*_*bind*_, a model based on only the binding conformation with lowest interaction energies displays RMSE and SDEP_CV_ close to the most predictive model (which includes maximally 6 poses per ligand, Table C in S1 File), with values of 4.2 kJ mol^-1^ and 4.4 kJ mol^-1^, respectively. However, it should also be noted that using a single binding pose will still require running multiple simulations to score and rank the simulations based on MD. Therefore, the final model in Fig 1 is as compute-efficient as a model based on a single highest-ranked pose only.

A previous CYP 1A2 LIE model was published by Vasanthanathan *et al*. using 8 compounds as training set and a single protein simulation per ligand to evaluate Δ*V* ^*Ele*^ and Δ*V* ^*VdW*^ in Eq 2 [27]. While this LIE model showed a comparable accuracy in reproducing Δ*G*_*bind*_ (with a reported RMSE of 3.7 kJ mol^-1^), Vasanthanathan found that differences in the electrostatic contributes among the training set did not affect the affinity toward CYP 1A2 (*β* = 0) and an offset parameter *γ* was introduced. Despite differences in electrostatic energy contributes, all the eight ligands presented polar groups and were found to undertake hydrogen bonds with water or amino acids within the catalytic site. In the current study the extension of the dataset to lipophilic compounds with no hydrogen bond donors or acceptors allowed a more extensive exploration of the ligand-protein interaction space, indicating a significant role for the electrostatic interactions to the binding affinity towards CYP 1A2 (*β* = 0.267, Fig 1) and leading to an affinity model in which the offset parameter *γ* was set to zero.

### Assessing the reliability of LIE predictions

The availability of a relatively large set of experimental inhibition data allows us to create a consistent external test set. This makes it possible to perform a more extensive validation of the model and to evaluate an approach to estimate the quality of predictions of a LIE model. Because LIE models include empirically derived parameters, it is reasonable that a prediction for a query compound can be considered reliable only if it is inferred from a region of information defined by the protein-ligand interactions that have been already explored during training of the model. Analysis of this space is required to identify the sources for the relatively large deviations of some of the predicted test-set affinities from experiment, and is performed here subsequently in terms of *(i)* the chemical similarity between the test set and training compounds, *(ii)* average protein-ligand interaction energies, and *(iii)* ligand-protein interactions during simulation.

### Chemical similarity analysis

**Fig 2:**
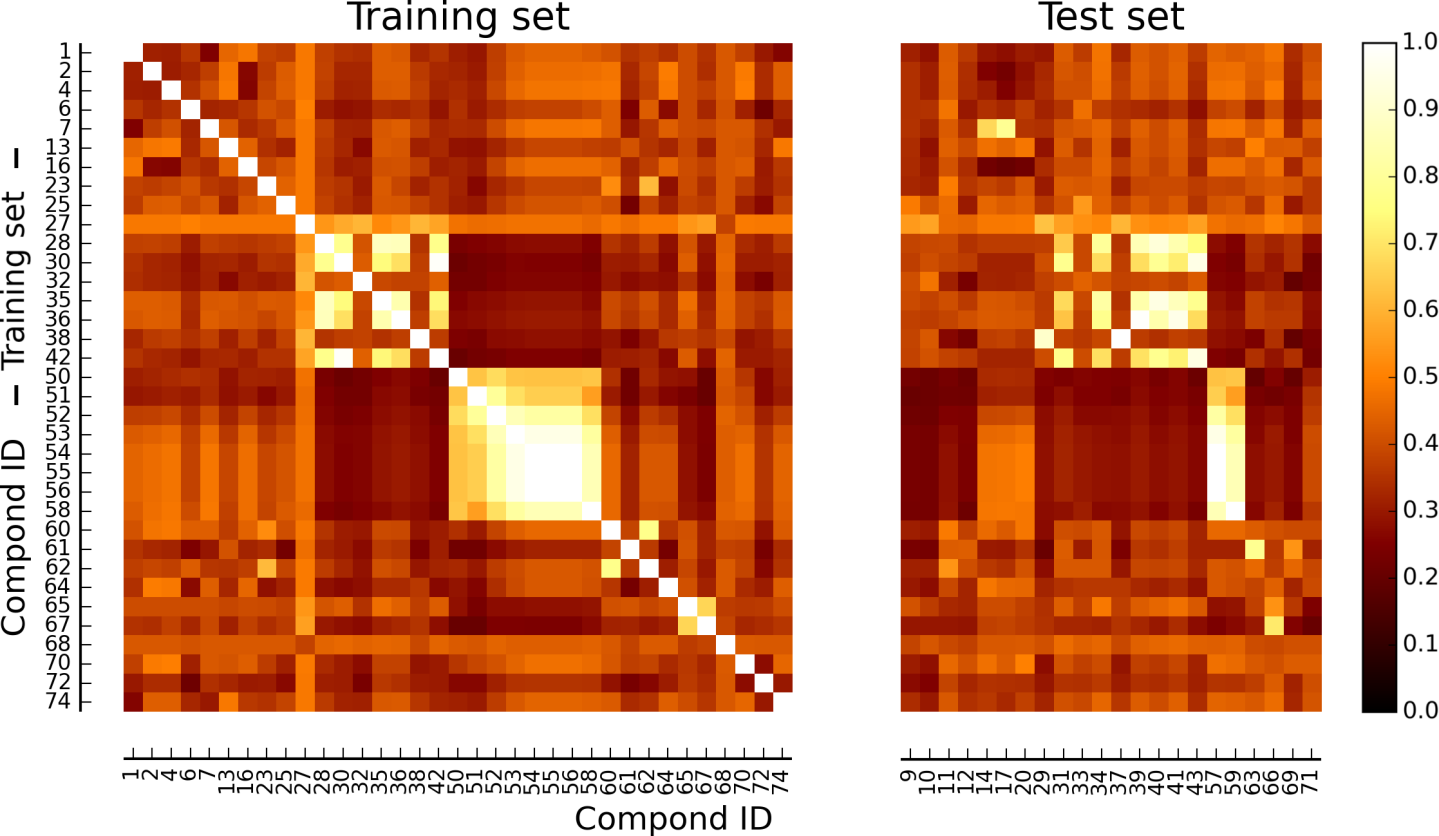
Similarity matrix of the data set. Heat map of the compounds included in the training and test set, colored according to percent similarity expressed in terms of Tanimoto scores (TSs) between pairs of structural fingerprints (white = 100% similarity (TS = 1.00); black = 0% similarity (TS = 0.00)).

Chemical similarity among the training set was evaluated as TS between each pair of molecules, represented as molecular fingerprints (Fig 2). Average similarity scores among the elements of the training set was between 0.32 (compound 72) and 0.50 (compound 27). Furthermore, every training compound was found to have a TS higher than 0.45 with at least one of the other compounds of the training set. Molecules from the test set were also compared to those of the training set. Average similarity scores ranged between 0.31 (compound 10) and 0.48 (compound 57), and only compounds 12 and 71 showed for all TSs values lower than 0.45 when compared to the training set molecules.

The relatively low average TS obtained among the training set compounds confirmed a significant diversity in terms of chemical structures. An analogous similarity/diversity can be observed when comparing the test set to the training set compounds. Within the test set, compounds 12 and 71 (Table A in S1 File) were considered to be outlier in this structural analysis because of their low TS obtained when compared to the most similar compound within the training set.

The error in prediction for the two compounds was respectively -6.4 and 5.6 kJ mol^-1^ (Table 4), suggesting that structural analysis based on fingerprint distance is able to identify few compounds with relatively high errors in prediction when compared to the SDEP_CV_. However, the chemical similarity analysis was not able to detect any of the other LIE predictions with significant deviation from experiment (Fig 1).

### Average interaction energy distributions

From a statistical point of view, LIE models can be considered as linear regressions in which average ensemble interaction energies Δ*V* ^*Ele*^ and Δ*V* ^*VdW*^ are used as variables (where Δ*V* ^*Ele*^ and Δ*V* ^*VdW*^ are obtained from MD simulations of the compounds in solvent and bound to the protein in the different binding configurations obtained from docking). With this in mind, it can be assumed that a prediction will not be accurate if the MD-averaged (differences in) interaction energies as obtained for a query compound do not belong to the distribution of the averaged interaction energies employed to train the model (*i*.*e*., as obtained from the MD simulations involving the training compounds) [15].

**Fig 3:**
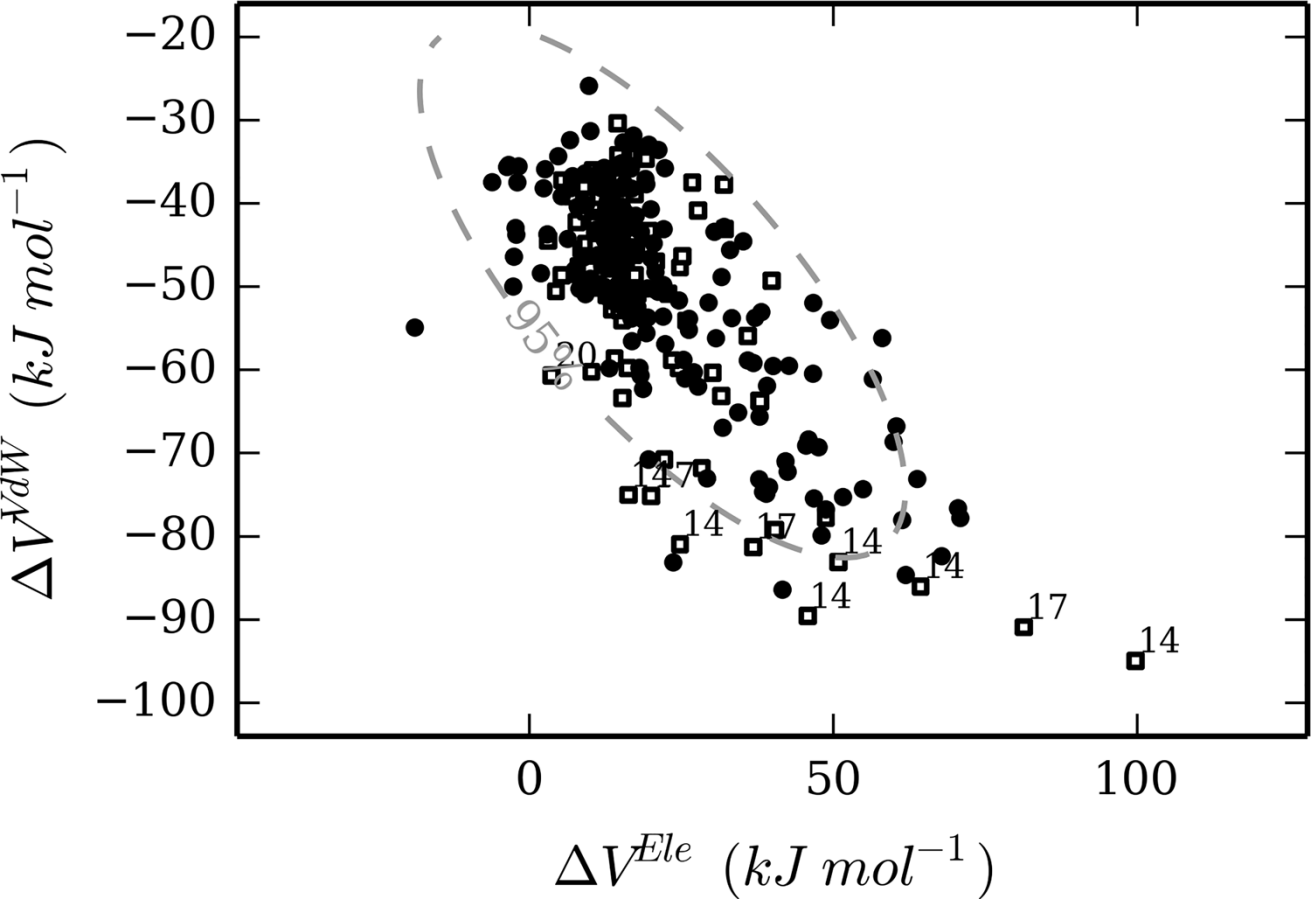
Distribution of Δ*V* ^*Ele*^ and Δ*V* ^*VdW*^ values (Eq 2) for training-set (black circles) and test-set (white squares) MD simulations. The dashed line represents the confidence for the 95 percentile of the training set distribution. The simulations from the test set that are not comprised in this interval are labeled according to the corresponding compound ID.

The Mahalanobis distance from the center of the distribution of training set values for Δ*V* ^*Ele*^ and Δ*V* ^*VdW*^ was measured for each simulation of the test set. Compounds 14, 17, 20 were found to show at least one simulation for which the Mahalanobis distance was larger than the value expected for the 95 percentile of the distribution of the training set (Fig 3). Interestingly, the three compounds showed errors in affinity prediction of 8.0, 7.8, and 7.0 kJ mol^-1^, respectively (Table 4).

Whereas these three outliers were not identified by our chemical similarity analysis, the evaluation of the distance of each simulation of the query compounds from the center of the distribution of training set simulations, in the space provided by their MD-averaged interaction energies, efficiently identified three LIE predictions with too large deviation from the observed inhibitory potency. However, several other compounds with significant deviations between calculated and observed values for Δ*G*_*bind*_ were not detected (Fig 1 and Table 4).

### Per-residue analysis of ligand-protein interactions

To evaluate the space of the electrostatic and steric interactions between the protein and compounds of the training set simulations, a per-residue decomposition was performed of the average nonbonded interaction energies (〈*V* ^*VdW*^_*lig-surr*_〉 or 〈*V* ^*Ele*^_*lig-surr*_〉) between the ligand and the amino acids lining the active site.

A decomposition of the electrostatic energy contributes indicated that the main residues involved in the interaction with the ligands were Thr_118_, Asn_312_, Asp_313_, Thr_124_, Asp_320_, and the water molecules in the active site (Figure A in S1 File), while Phe_226_, Ala_317_, Gly_316_, Asp_313_, Phe_260_, Ile_117_, Phe_125_, Leu_497_, and water represented the residues with the highest average van der Waals interaction energies 〈*V* ^*VdW*^_*lig-surr*_〉 contributes (Figure B in S1 File). These observations overlap with previous findings: analysis of the CYP 1A2 crystal structure [20] indicated that Thr_118_ and Thr_124_ lie in a hydrophilic region that is considered important for the binding of polar substrates, while Phe_226_, Ala_317_, and Gly_316_ form a planar surface that is important for recognizing flat molecules. The crystal structure showed also that Phe_125_ contributes to tight binding of the co-crystallized alpha-naphthoflavone [20]. Additionally, a recent computational study by Yang *et al*. [74] suggested Asn_312_, Asp_313_, and Phe_260_ to be critical for binding of acetominophen to CYP 1A2.

**Fig 4:**
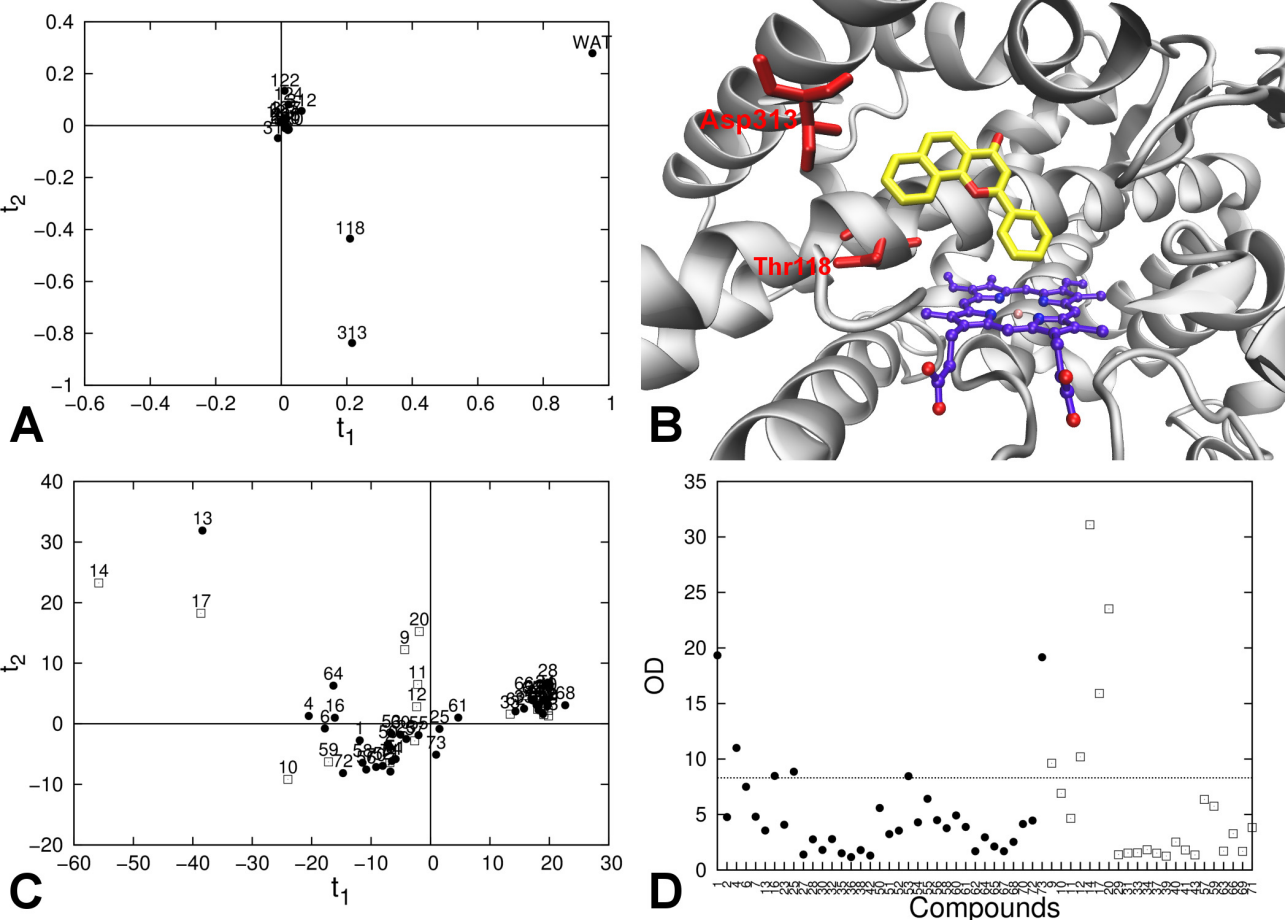
Per-residue decomposition analysis of the electrostatic interaction energies between the ligand and its surrounding in the protein-ligand simulations. (A) PCA loading plot for training-set electrostatic interaction energies; (B) Active site of CYP 1A2 from the crystallographic structure; heme group (purple carbon atoms), co-crystallized ligand α-naphthoflavone (yellow carbon atoms), and amino acids with high loading on the first two PCs (in red) are explicitly represented. (C) PCA score plot for the training-set (black circles) and test-set (white squares) compounds for the first two PCs. (D) Orthogonal distance (OD) of the compounds of the training set (black circles) and test set (white squares) from the model with 2 PCs. The dashed horizontal line represents the critical orthogonal distance, calculated for the training-set distribution.

PCA on the decomposed electrostatic contributes for each compound of the training set led to identification of two principal components. The first component accounted for 71.3% of the per-residue energy contributes variability, and was dominated by the interaction with the water molecules present in the catalytic site. Interactions with Asp_313_ and Thr_118_ dominated the second component, which explains 15.2% of the overall variability (Fig 4, panels A and B). The plot of the first two PC scores showed that the compounds are clustered in three groups: (1) compounds with non-polar groups (compounds 27, 28, 30, 32, 35, 36, 38, 42, 62, 65, 67, 68, 29, 31, 33, 34, 37, 39, 40, 41, 43, 63, 66, 69) were clustered in a small region with positive scores on the first component, and scores close to 0 on the second; (2) compounds 13, 14 and 17 (bearing a high number of hydrogen bond donor groups or acceptors) showed high scores on both principal components; and (3) all other compounds were homogeneously distributed around the origin (Fig 4, panel C). Score and orthogonal outliers in the test set were detected in the rotated space (Fig 4, panels C and D). Compounds 9, 12 and 20 were orthogonal outliers, while 14 and 17 were both orthogonal and score outliers. Errors in prediction for these compounds were respectively -11.6, -6.4, 7.0, 8.0, and 7.8 kJ mol^-1^ (Table 4).

**Fig 5:**
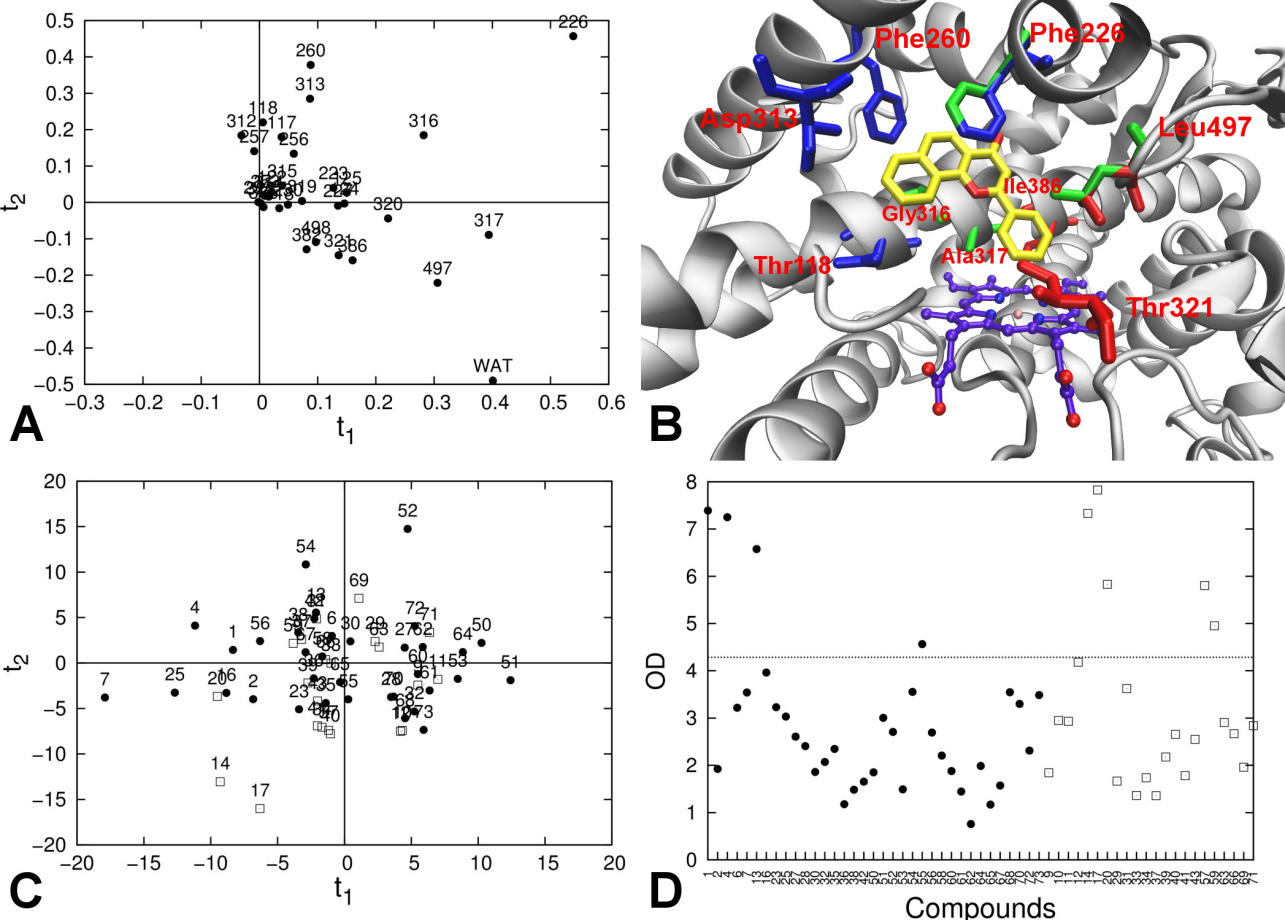
Per-residue decomposition analysis of the van der Waals interaction energies between the ligand and its surrounding in the protein-ligand simulations. (A) PCA loading plot for training-set van der Waals interaction energies; (B) Active site of CYP 1A2 from the crystallographic structure; heme group (purple carbon atoms), the co-crystallized ligand α-naphthoflavone (yellow carbon atoms), and amino acids with high loadings in the PCA are explicitly represented. Residues with high positive loadings on the first PC are depicted in green; Residues with high loadings on the second component are also represented, both for positive (blue) and negative values (red). (C) PCA score plot for the training-set (black circles) and test-set (white squares) compounds for the first two PCs. (D) Orthogonal distance (OD) of the compounds of the training set (black circles) and test set (white squares) from the model with 4 PCs. The dashed horizontal line represents the critical orthogonal distance, calculated for the training-set distribution.

In an analogue PCA performed on the per-residue decomposition of the average van der Waals interaction energies 〈*V* ^*VdW*^_*lig-surr*_〉, 89.5% of the multivariate variability was accounted by 4 principal components. High loading on the first principal component (45.7% of the overall variability) was shown by the interaction with residues in the center of the catalytic site (Gly_316_ and Ala_317_ in helix I, Phe_226_ and Leu_497_ on the opposite side; Fig 5, panels A and B). On the second principal component (21.6% of the overall variability), a positive loading was provided by interaction with residues in the portion of the pocket delimited by the helices F, G, and I (residues Phe_226_, Phe_260_, Asp_313_, Thr_118_) while opposite contributes were provided by the interaction with residues on the other side of the catalytic site (Water, Leu_497_, Ile_386_, Thr_321_; Fig 5, panels A and B). Components 3 and 4 explained 13.2% and 9.0% of the multivariate variability, respectively, but their meaning is more uncertain. Analysis of the scores showed a homogeneous distribution of the compounds, except for compounds 14 and 17, which were found to be score outliers (Fig 5, panel C). Compounds 14 and 17 appeared to be also orthogonal outliers in the evaluation of the residuals, together with compounds 20, 57, and 59 (Fig 5, panel D). Errors in prediction for these compounds were 8.0, 7.8, 7.0, 5.4, and 5.5 kJ mol^-1^, respectively.

The interaction of a ligand with its (off-)target protein is depending on both steric and electrostatic properties of the binding site. Within a large and malleable binding pocket such as the catalytic site of several drug-metabolizing CYPs, a ligand can bind in different conformations and in different topological parts of the pocket (*i*.*e*., with different electrostatic and steric properties). While the averaged electrostatic and van der Waals ensemble energies are typically unable to give fine details about the topology of the interactions, analysis of the nonbonded interaction energy contributes from each residue can provide more details about how ligands interact with the catalytic site. The per-residue decomposition of the interaction energies was used to identify different areas of the catalytic site that are considered important for the recognition of CYP 1A2 substrates and inhibitors, and PCA on these data was able to characterize the regions of the catalytic site that are critical for binding of specific ligands. Moreover, we could use this analysis to identify query compounds presenting a pattern of interactions that was not properly sampled by the training set (orthogonal outliers), or for which the interactions with single residues were unusually strong, as compared to what was observed within the training set (score outliers). Both groups of molecules showed absolute errors in affinity prediction between 5.4 and 11.6 kJ mol^-1^, which are considerably larger when compared to the errors obtained for the training set (Table 3).

### Combined approach to analyse the prediction reliability

**Fig 6:**
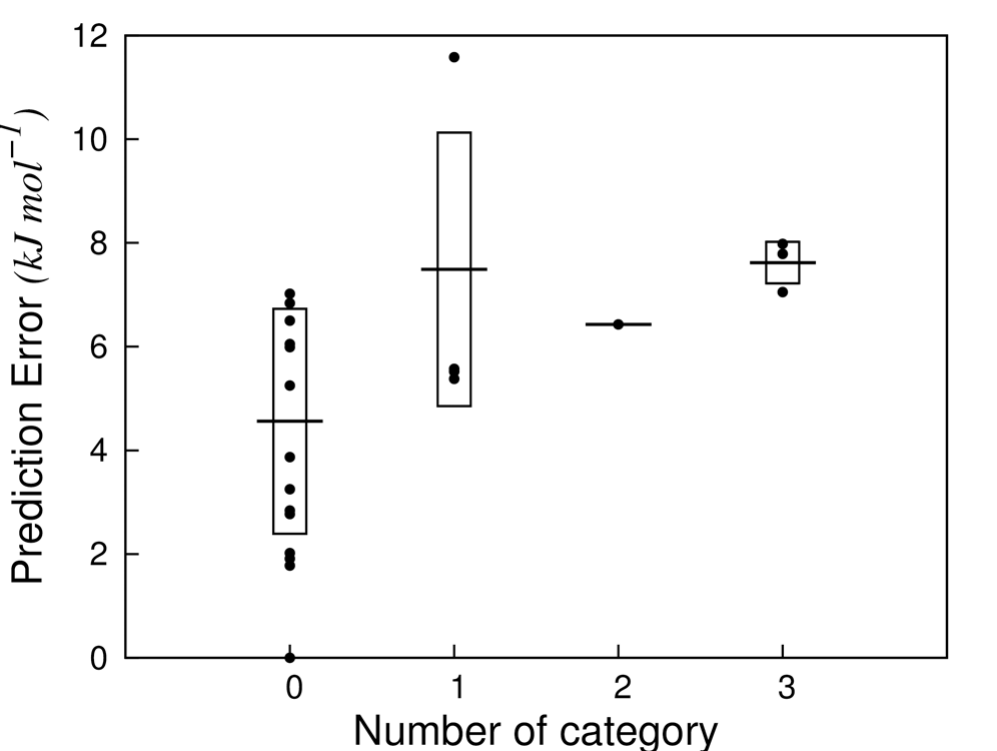
Prediction errors obtained for the external test set compounds. The compounds were grouped in a category according to the number of occurrences in which they were found to be an outlier according to analyses (A)-(D) in Table 4. Horizontal lines represent the standard error (SDEP) for a given category, while the boxes represent the standard deviation around this average.

Similar to the ADAN approach of Carrió *et al*. for traditional QSAR methods [17], we adopted a *combined* approach in which the analyses presented above were used as criteria to assess the reliability of LIE predictions. These criteria were based on (A) chemical similarity analysis, (B) average interaction energy distribution analysis, (C) per-residue analysis of the ligand-protein electrostatic interactions, and (D) per-residue analysis of the ligand-protein van der Waals interactions. According to the total number of analyses (0 to 4) in which they were found to be outliers, the query compounds were thus classified in 5 categories (Fig 6). 14 test set compounds were found to be outlier in none of the analyses (category 0), and showed a SDEP of 4.6 kJ mol^-1^ between calculated and experimental Δ*G*_*bind*_, Fig 6. Four compounds (9, 57, 59, 71) were outliers in one analysis (category 1), for which the SDEP was 7.5 kJ mol^-1^. One compound (12), with an error in prediction of 6.4 kJ mol^-1^) was outlier in two analyses, while three compounds were outlier in three analyses and showed the highest SDEP of 7.6 kJ mol^-1^ (14, 17, 20). None of the compounds of the test set was found to be outlier in all analyses.

Considering the obtained results, compounds that were outlier in an increasing number of analyses were found to show larger errors in prediction. Moreover, the compounds with no deviations showed a SDEP similar to the one obtained during LOO-CV of the model (4.6 vs. 4.3 kJ mol^-1^), indicating that our combined analysis on the nature of the ligands and their interactions with the protein of interest can be used for assessing the reliability of predictions by protein-structural and -dynamical based models.

## Conclusions

An iterative LIE model for predicting CYP 1A2 binding affinities was presented. A dataset of *IC*_*50*_ values for 73 compounds characterized by a large chemical diversity was collected from three different literature sources, and the experimental uncertainty was estimated by measuring the inhibitory potency in-house for a sample of compounds from each source under the same conditions. The RMSD between in-house measured *IC*_*50*_ values and literature values of 3.3 kJ mol^-1^ (with a maximum variation of 6.9 kJ mol^-1^) was used as limit to identify possibly overfitted models.

From the dataset, 35 compounds that covered a broad range of chemical diversity (with an average Tanimoto Score among pairs of compounds of 0.39) were used to calibrate our LIE model, which showed high correlation between calculated and observed values for the binding free energy (r^2^ = 0.68; q^2^_CV_ = 0.66). Errors in regression (RMSE) and in prediction in cross validation (SDEP_CV_) were comparable to the uncertainty in the experimental data (RMSE = 4.1 kJ mol^-1^; SDEP_CV_ = 4.3 kJ mol^-1^), indicating that the MD-based approach was able to properly address the protein and ligand flexibility that are crucial in modeling CYP450-ligand interactions.

Compounds that were not included in the training set were used to evaluate the predictivity of the model. Since we provided a parameterized model, the inhibitory potency for a query (test-set) compound can only be accurately predicted if it shows sufficient similarity with the elements of the training set that determine the model. Assuming that such similarity within a structure-based model depends on both the compound and the specific interactions it undertakes with the (off-)target protein, we proposed a set of analyses to evaluate the different elements playing a role in determining a LIE-based prediction. Analysis of the chemical structure of the ligands only was very limited in detecting test-set compounds with large deviations in prediction. By also taking into account the protein-ligand interactions and by combining results from multiple analyses in a single score, it was possible to obtain good estimation of the prediction reliability. Predictions that were found to be most reliable (*i*.*e*., for which the compounds were not found to be outlier in any of the applied analyses) showed a SDEP of 4.6 kJ mol^-1^, which is comparable to the SDEP_CV_ obtained, while predictions for groups of compounds with decreasing level of reliability showed a SDEP of 7.5, 6.4, and 7.6 kJ mol^-1^, respectively.

In this work we presented a model for CYP 1A2 binding affinity calculation using an approach based on a highly automatable and scalable protocol that has proved to be applicable in predicting binding affinities for compounds characterized by broad chemical diversity. Additionally, we provided an innovative method that was able to estimate the reliability of single MD-based predictions, thereby efficiently including information on the ligand-protein interactions. Considering the continuous increment in accessibility of computational power, the comprehensive method we proposed here represents a promising alternative to traditional quantitative structure-activity(/property) relationship models for a vast range of biological (off-)targets for which a 3D structure is available and in which protein structural and/or dynamical information is crucial to model the interaction with ligands and predict the property or properties of interest.

## Supporting Information

S1 File Per-residue decomposition of the average electrostatic interaction energies 〈*V* ^*Ele*^〉 (Figure A). Per-residue decomposition of the average Van der Waals interaction energies 〈*V* ^*VdW*^〉 **(Figure B)**. Structures and *IC*_*50*_ values from literature for the data set of compounds **(Table A)**. Reacting chemical groups in (quasi-)irreversible CYP inhibitors **(Table B)**. Overview of the average electrostatic 〈*V* ^*Ele*^〉 and van der Waals 〈*V* ^*VdW*^〉 interaction energies **(Table C)**.
(PDF)

## Abbreviations

PCA: principal component analysis
CYP: Cytochrome P450
CYP 1A2: Cytochrome P450 1A2
MD: molecular dynamics
LIE: linear interaction energy
TS: Tanimoto score
