# Supplementary material for: Linear Interaction Energy Based Prediction of Cytochrome P450 1A2 Binding Affinities with Reliability Estimation
Source: PLoS One. 2015 Nov 9;10(11):e0142232. doi: 10.1371/journal.pone.0142232 (PMC4638363; doi:10.1371/journal.pone.0142232)
Supplement: S1 File — Per-residue decomposition of the average Van der Waals interaction energies 〈V VdW〉 (Figure B). Structures and IC 50 values from literature for the data set of compounds (Table A). Reacting chemical groups in (quasi-)irreversible CYP inhibitors (Table B). Overview of the average electrostatic 〈V Ele〉 and van der Waals 〈V VdW〉 interaction energies (Table C). (PDF) [file pone.0142232.s001.pdf]

**S1 Supporting Information.** Per-residue decomposition of the average electrostatic interaction energies $\langle V^{Ele} \rangle$ **(Figure A)**. Per-residue decomposition of the average Van der Waals interaction energies $\langle V^{VdW} \rangle$ **(Figure B)**. Structures and $IC_{50}$ values from literature for the data set of compounds **(Table A)**. Reacting chemical groups in (quasi-)irreversible CYP inhibitors **(Table B)**. Overview of the average electrostatic $\langle V^{Ele} \rangle$ and van der Waals $\langle V^{VdW} \rangle$ interaction energies **(Table C)**.

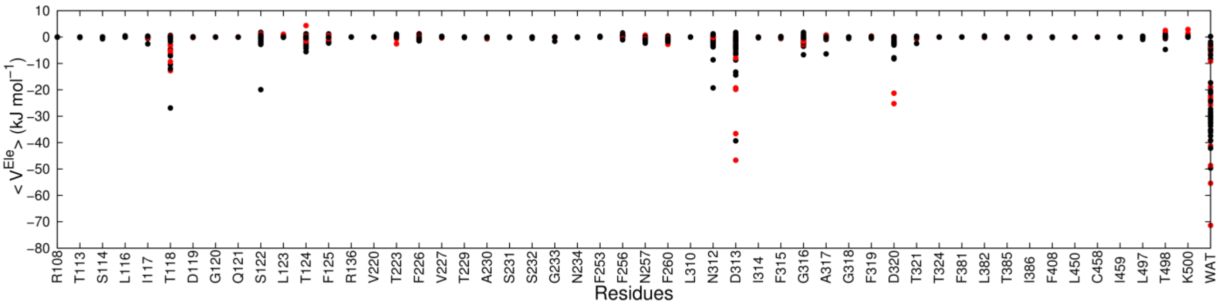


**Figure A. Per-residue decomposition of the average electrostatic interaction energy <$V^{Ele}$>.** Interaction energies are reported between CYP 1A2 active site residues and training (black) and test set (red) compounds during the multiple MD simulations of the CYP 1A2-ligand complex performed per compound.

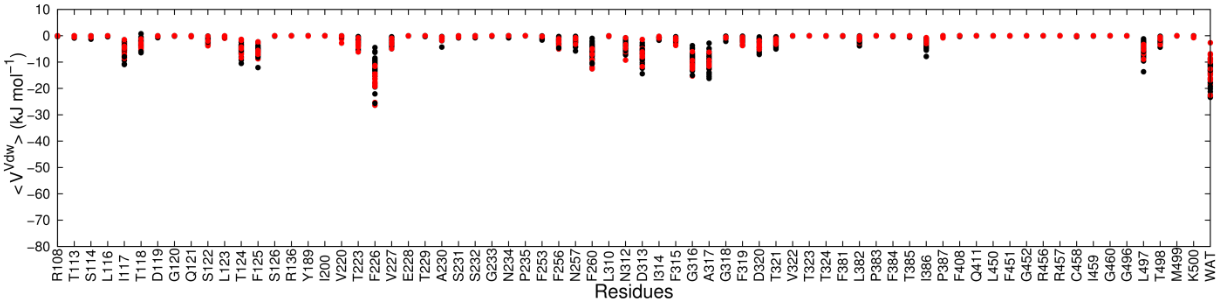


**Figure B. Per-residue decomposition of the average van der Waals interaction energy <$V^{VdW}$>.** Interaction energies are reported between CYP 1A2 active site residues and training (black) and test set (red) compounds during the multiple MD simulations of the CYP 1A2-ligand complex performed per compound.

**Table A. Structures and $IC_{50}$ values from literature for the data set of compounds.** Compounds are classified into LIE model training (Train) and test (Test) set. Inhibitors that are predicted to not be competitive are also reported (NC).

| ID | Structure | Molecule | $IC_{50}$ (μM) | Source | Classification |
|---|---|---|---|---|---|
| 1 | 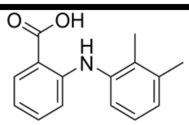 | Mefenamic Acid | 13.98 | 1 | Train |
| 2 | 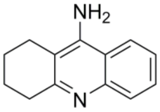 | Tacrine | 5.20 | 1 | Train |
| 3 | 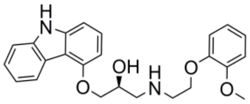 | Carvedilol | 5.10 | 1 | NC |
| 4 | 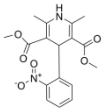 | Nifedipine | 5.74 | 1 | Train |
| 5 | 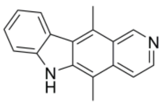 | Ellipticine | 0.11 | 2 | NC |
| 6 | 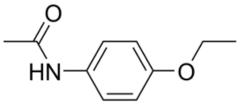 | Phenacetin | 28.00 | 2 | Train |
| 7 | 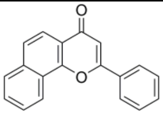 | alpha-Naphthoflavone | 0.08 | 2 | Train |
| 8 | 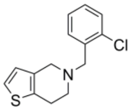 | Ticlopidine | 22.43 | 2 | NC |
| 9 | 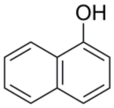 | 1-naphthol | 3.2 | 3 | Test |
| 10 | 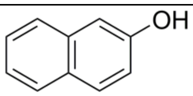 | 2-naphthol | 17 | 3 | Test |
| 11 | 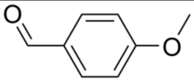 | p-methoxybenzaldehyde | 270 | 3 | Test |

| | | | | | |
|---|---|---|---|---|---|
| 12 | 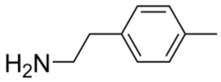 | p-tolylethylamine | 14 | 3 | Test |
| 13 | 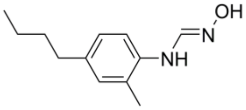 | HET-0016 | 1.09 | 1 | Train |
| 14 | 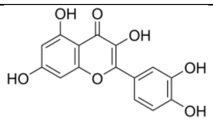 | Quercetin | 5.52 | 1 | Test |
| 15 | 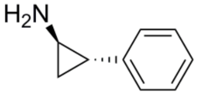 | Tranylcypromine | 10.42 | 1 | NC |
| 16 | 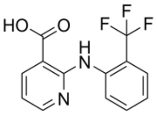 | Niflumic Acid | 11.59 | 1 | Train |
| 17 | 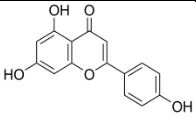 | Naringenin | 12.35 | 1 | Test |
| 18 | 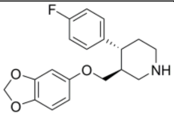 | Paroxetine | 23.46 | 1 | NC |
| 19 | 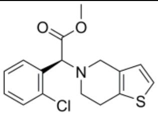 | Clopidogrel | 24.15 | 1 | NC |
| 20 | 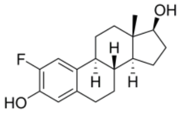 | 2-Fluoroestradiol | 26.75 | 1 | Test |
| 21 | 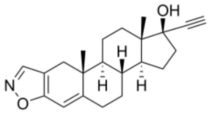 | Danazol | 45.32 | 1 | NC |
| 22 | 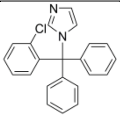 | Clotrimazole | 5.83 | 1 | NC |
| 23 | 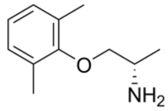 | Mexiletine | 1.84 | 2 | Train |

| | | | | | |
|---|---|---|---|---|---|
| 24 | 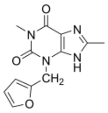 | Furafylline | 1.50 | 2 | NC |
| 25 | 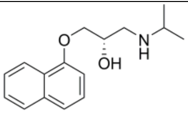 | Propranolol | 3.84 | 2 | Train |
| 26 | 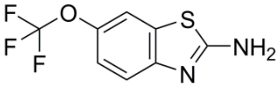 | Riluzole | 15.8 | 2 | NC |
| 27 | 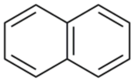 | Naphthalene | 700 | 3 | Train |
| 28 | 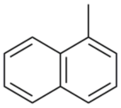 | 1-Methylnaphthalene | 110 | 3 | Train |
| 29 | 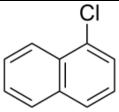 | 1-Chloronaphthalene | 50 | 3 | Test |
| 30 | 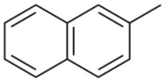 | 2-Methylnaphthalene | 120 | 3 | Train |
| 31 | 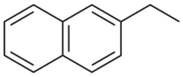 | 2-Ethylnaphthalene | 19 | 3 | Test |
| 32 | 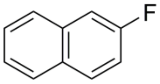 | 2-Fluoronaphthalene | 49 | 3 | Train |
| 33 | 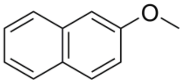 | 2-Methoxynaphthalene | 13 | 3 | Test |
| 34 | 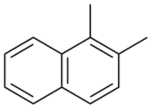 | 1,2-Dimethylnaphthalene | 5.5 | 3 | Test |
| 35 | 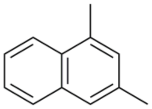 | 1,3-Dimethylnaphthalene | 7.9 | 3 | Train |
| 36 | 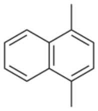 | 1,4-Dimethylnaphthalene | 3.6 | 3 | Train |

| | | | | | |
|---|---|---|---|---|---|
| 37 | 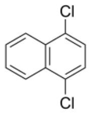 | 1,4-Dichloronaphthalene | 2.3 | 3 | Test |
| 38 | 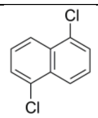 | 1,5-Dichloronaphthalene | 14 | 3 | Train |
| 39 | 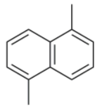 | 1,5-Dimethylnaphthalene | 42 | 3 | Test |
| 40 | 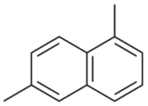 | 1,6-Dimethylnaphthalene | 25 | 3 | Test |
| 41 | 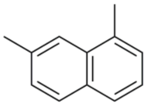 | 1,7-Dimethylnaphthalene | 7.5 | 3 | Test |
| 42 | 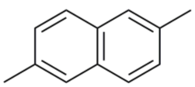 | 2,6-Dimethylnaphthalene | 52 | 3 | Train |
| 43 | 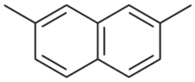 | 2,7-Dimethylnaphthalene | 65 | 3 | Test |
| 44 | 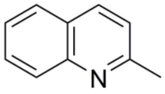 | Quinaldine | 40 | 3 | NC |
| 45 | 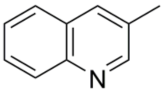 | 3-Methylquinoline | 13 | 3 | NC |
| 46 | 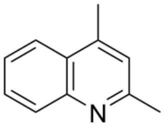 | 2-4-Dimethylquinoline | 2.4 | 3 | NC |
| 47 | 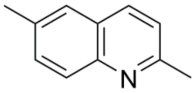 | 2,6-Dimethylquinoline | 3.3 | 3 | NC |
| 48 | 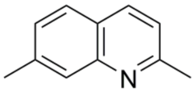 | 2,7-Dimethylquinoline | 4.4 | 3 | NC |

| | | | | | |
|---|---|---|---|---|---|
| 49 | 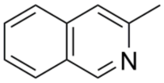 | 3-Methylisoquinoline | 21 | 3 | NC |
| 50 | 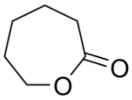 | ε-Caprolactone | 40,000 | 3 | Train |
| 51 | 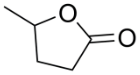 | γ-Valerolactone | 15,000 | 3 | Train |
| 52 | 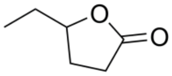 | γ-Caprolactone | 9,900 | 3 | Train |
| 53 | 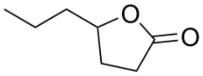 | γ-Heptalactone | 4,500. | 3 | Train |
| 54 | 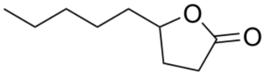 | γ-Nonanoiclactone | 310 | 3 | Train |
| 55 | 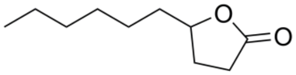 | γ-Decanolactone | 230 | 3 | Train |
| 56 | 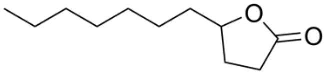 | γ-Undecanoiclactone | 72 | 3 | Train |
| 57 | 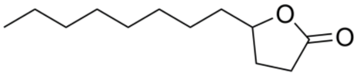 | γ-Dodecanolactone | 58 | 3 | Test |
| 58 | 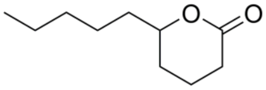 | δ-Decanolactone | 230 | 3 | Train |
| 59 | 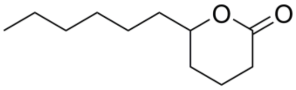 | δ-Undecanolactone | 72 | 3 | Test |
| 60 | 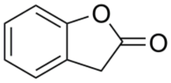 | 2-Coumarone | 260 | 3 | Train |
| 61 | 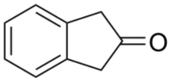 | 2-Indanone | 80 | 3 | Train |
| 62 | 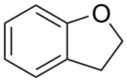 | 2,3-Dihydrobenzofuran | 1,200 | 3 | Train |

| | | | | | |
|---|---|---|---|---|---|
| 63 | 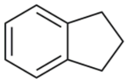 | Indan | 550 | 3 | Test |
| 64 | 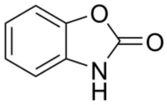 | 2-Benzoxazolinone | 370 | 3 | Train |
| 65 | 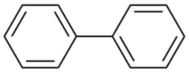 | Biphenyl | 160 | 3 | Train |
| 66 | 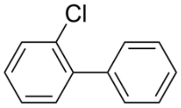 | 2-Chlorobiphenyl | 230 | 3 | Test |
| 67 | 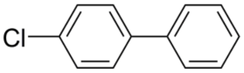 | 4-Chlorobiphenyl | 49 | 3 | Train |
| 68 | 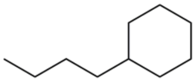 | Butylcyclohexane | 55 | 3 | Train |
| 69 | 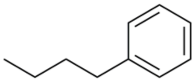 | Butylbenzene | 3,700 | 3 | Test |
| 70 | 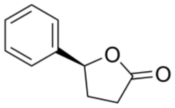 | γ-Phenyl-γ-butyrolactone | 2,300 | 3 | Train |
| 71 | 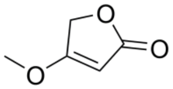 | 4-Methoxyfuran-2-(5H)-one | 40,000 | 3 | Test |
| 72 | 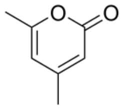 | 4,6-Dimethyl-α-pyron | 4,500 | 3 | Train |
| 73 | 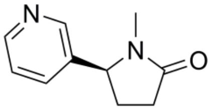 | Cotinine | 5,400 | 3 | Train |

Source 1: Bayer HealthCare databases for CYP inhibition, made available within *eTOX* consortium. Source 2: Cohen, L.; Remley, M.; Raunig, D.; Vaz, A. In Vitro Drug Interactions of Cytochrome P450: An Evaluation of Fluorogenic to Conventional Substrates. Drug Metab. Dispos. 2003, 31, 1005–1015. Source 3: Korhonen, L. E.; Rahnasto, M.; Mähönen, N. J.; Wittekindt, C.; Poso, A.; Juvonen, R. O.; Raunio, H. Predictive Three-dimensional Quantitative Structure-activity Relationship of Cytochrome P450 1A2 Inhibitors. J. Med. Chem. 2005, 48, 3808–3815.

**Table B. Reacting chemical groups in (quasi-)irreversible CYP inhibitors.**

| Chemical Group | Mechanism of inhibition* |
|---|---|
| Aromatic Nitrogens | Quasi-irreversible inhibition |
| Isothiocyanates | Mechanism-based inhibition |
| Thioureas | Mechanism-based inhibition |
| Thiophenes | Mechanism-based inhibition |
| Thiols | Mechanism-based inhibition |
| Thiocyanate | Mechanism-based inhibition |
| Clorinated groups | Mechanism-based inhibition |
| Alkenes/Alkynes | Mechanism-based inhibition |
| Tertiary amines in heterocyclic rings | Mechanism-based inhibition |
| Furanocumarines | Mechanism-based inhibition |
| Furanopyridins | Mechanism-based inhibition |
| Methylene dioxycompounds | Mechanism-based inhibition |
| Amines | Mechanism-based inhibition |
| Hydrazines | Mechanism-based inhibition |
| Dihydropyridines | Mechanism-based inhibition |
| Dyhydroquinolines | Mechanism-based inhibition |
| Aldehydes | Mechanism-based inhibition |

*From: Correia, M. A., Ortiz de Montellano P. R. Inhibition of Cytochrome P450 Enzymes. In Cytochrome P450: Structure, Mechanism, and Biochemistry; 3e; Ortiz de Montellano, P. R., Ed; Kluwer Academic / Plenum Publishers, New York, 2005.

**Table C. Overview of average electrostatic $\langle V^{Ele} \rangle$, and van der Waals $\langle V^{VdW} \rangle$ interaction energies.** The energies and their standard deviations are obtained from MD for each ligand, simulated in solvent and in all the orientations obtained from docking and clustering. Relative weights $W_i$ of the energy for each simulation contributing to the LIE model (maximum 6 poses, $\alpha$=0.587, $\beta$=0.267) are also reported.

| ID | Solvent | | Protein-bound | | | |
|---|---|---|---|---|---|---|
| | $\langle V^{Ele} \rangle$ | $\langle V^{VdW} \rangle$ | **Pose** | $\langle V^{Ele} \rangle$ | $\langle V^{VdW} \rangle$ | $W_i$ |
| 1 | -110.1 ± 21.4 | -77.9 ± 11.2 | 1 | -71.6 ± 11.5 | -152.6 ± 10.0 | 0.32 |
| | | | 2 | -53.6 ± 17.8 | -139.0 ± 11.7 | 0.00 |
| | | | 3 | -91.9 ± 18.3 | -137.7 ± 11.2 | 0.09 |
| | | | 4 | -50.1 ± 11.1 | -146.6 ± 9.6 | 0.01 |
| | | | 5 | -80.8 ± 10.8 | -150.9 ± 9.7 | 0.58 |
| 2 | -73.8 ± 18.1 | -72.0 ± 10.2 | 1 | -46.1 ± 14.8 | -134.0 ± 9.8 | 0.15 |
| | | | 2 | -48.5 ± 14.6 | -130.8 ± 9.4 | 0.09 |
| | | | 3 | -39.5 ± 14.0 | -137.1 ± 9.2 | 0.16 |
| | | | 4 | -48.2 ± 12.6 | -133.1 ± 8.8 | 0.15 |
| | | | 5 | -42.0 ± 11.7 | -139.0 ± 9.1 | 0.32 |
| | | | 6 | -36.0 ± 12.6 | -137.6 ± 9.3 | 0.12 |
| 4 | -129.4 ± 20.2 | -101.1 ± 12.8 | 1 | -83.5 ± 13.5 | -169.5 ± 12.2 | 0.13 |
| | | | 2 | -81.9 ± 18.9 | -170.4 ± 11.5 | 0.13 |
| | | | 3 | -73.3 ± 20.3 | -165.0 ± 13.1 | |
| | | | 4 | -61.6 ± 15.2 | -183.5 ± 11.9 | 0.33 |
| | | | 5 | -83.9 ± 19.1 | -170.2 ± 12.2 | 0.16 |
| | | | 6 | -69.1 ± 13.5 | -167.9 ± 11.0 | 0.02 |
| | | | 7 | -68.1 ± 15.6 | -179.1 ± 12.6 | 0.24 |
| 6 | -92.2 ± 16.7 | -65.8 ± 10.0 | 1 | -62.7 ± 11.2 | -117.7 ± 9.4 | 0.12 |
| | | | 2 | -70.9 ± 11.3 | -116.4 ± 9.8 | 0.22 |
| | | | 3 | -45.5 ± 11.5 | -126.3 ± 9.2 | 0.15 |
| | | | 4 | -54.0 ± 14.2 | -118.9 ± 8.8 | 0.06 |
| | | | 5 | -58.8 ± 12.9 | -119.6 ± 9.9 | 0.13 |
| | | | 6 | -56.2 ± 13.6 | -124.7 ± 9.5 | 0.32 |
| 7 | -63.3 ± 14.5 | -96.8 ± 9.7 | 1 | -21.7 ± 10.5 | -183.2 ± 8.7 | 0.22 |
| | | | 2 | -44.6 ± 11.8 | -159.1 ± 9.7 | 0.01 |
| | | | 3 | -43.7 ± 9.1 | -167.6 ± 9.6 | 0.06 |
| | | | 4 | -32.6 ± 10.7 | -153.0 ± 9.9 | 0.00 |
| | | | 5 | -39.6 ± 9.2 | -179.9 ± 8.7 | 0.71 |
| 13 | -101.7 ± 29.8 | -72.7 ± 11.8 | 1 | -30.8 ± 13.5 | -150.5 ± 9.3 | 0.01 |

| | | | | | | |
|---|---|---|---|---|---|---|
| | | | 2 | -59.0 ± 19.2 | -132.2 ± 12.1 | 0.00 |
| | | | 3 | -83.4 ± 18.5 | -133.4 ± 11.4 | 0.07 |
| | | | 4 | -37.9 ± 16.9 | -145.8 ± 10.8 | 0.01 |
| | | | 5 | -31.2 ± 8.6 | -149.3 ± 8.8 | 0.01 |
| | | | 6 | -120.6 ± 19.8 | -127.6 ± 12.7 | 0.90 |
| 16 | -116.9 ± 31.3 | -74.7 ± 13.5 | 1 | -77.4 ± 18.4 | -148.7 ± 11.9 | 0.17 |
| | | | 2 | -65.3 ± 21.0 | -149.9 ± 11.4 | 0.06 |
| | | | 3 | -68.1 ± 23.6 | -151.4 ± 11.8 | 0.12 |
| | | | 4 | -77.9 ± 19.5 | -149.5 ± 11.5 | 0.22 |
| | | | 5 | -68.8 ± 15.0 | -154.5 ± 10.3 | 0.27 |
| | | | 6 | -79.1 ± 22.0 | -147.8 ± 12.2 | 0.16 |
| 23 | -63.8 ± 14.5 | -65.5 ± 9.3 | 1 | -41.7 ± 11.8 | -116.1 ± 10.1 | 0.07 |
| | | | 2 | -26.9 ± 17.0 | -124.7 ± 8.3 | 0.10 |
| | | | 3 | -24.7 ± 13.0 | -127.4 ± 8.5 | 0.16 |
| | | | 4 | -36.7 ± 10.7 | -125.8 ± 8.0 | 0.38 |
| | | | 5 | -41.4 ± 10.4 | -122.4 ± 8.6 | 0.29 |
| 25 | -94.2 ± 22.9 | -92.2 ± 11.8 | 1 | -39.3 ± 11.1 | -166.5 ± 10.1 | 0.07 |
| | | | 2 | -32.2 ± 11.0 | -176.9 ± 8.7 | 0.39 |
| | | | 3 | -47.3 ± 18.4 | -167.7 ± 9.8 | 0.23 |
| | | | 4 | -51.7 ± 12.9 | -164.4 ± 10.0 | 0.17 |
| | | | 5 | -52.0 ± 22.5 | -163.2 ± 11.5 | 0.13 |
| 27 | -22.7 ± 8.0 | -53.2 ± 6.2 | 1 | -11.3 ± 3.9 | -90.4 ± 6.1 | 0.52 |
| | | | 2 | -6.9 ± 4.4 | -88.5 ± 6.3 | 0.21 |
| | | | 3 | -3.6 ± 4.9 | -90.2 ± 6.6 | 0.22 |
| | | | 4 | -12.9 ± 7.0 | -79.0 ± 7.1 | 0.04 |
| 28 | -21.8 ± 7.5 | -59.4 ± 6.1 | 1 | -8.8 ± 4.2 | -101.2 ± 6.4 | 0.17 |
| | | | 2 | -6.3 ± 3.5 | -101.2 ± 6.1 | 0.13 |
| | | | 3 | -8.6 ± 4.0 | -103.1 ± 6.2 | 0.26 |
| | | | 4 | -8.6 ± 3.9 | -105.5 ± 6.3 | 0.45 |
| 30 | -22.4 ± 8.0 | -59.9 ± 6.5 | 1 | -7.7 ± 4.2 | -100.6 ± 7.0 | 0.16 |
| | | | 2 | -11.0 ± 5.1 | -96.3 ± 6.9 | 0.08 |
| | | | 3 | -6.8 ± 4.5 | -103.2 ± 7.0 | 0.26 |
| | | | 4 | -7.1 ± 3.5 | -104.4 ± 6.0 | 0.35 |
| | | | 5 | -6.5 ± 4.7 | -101.1 ± 6.9 | 0.15 |
| 32 | -19.4 ± 7.4 | -55.0 ± 6.2 | 1 | -10.1 ± 4.9 | -95.2 ± 6.9 | 0.29 |
| | | | 2 | -10.2 ± 4.3 | -91.4 ± 6.9 | 0.12 |
| | | | 3 | -11.5 ± 5.2 | -95.4 ± 6.7 | 0.36 |
| | | | 4 | -12.5 ± 4.9 | -93.2 ± 6.9 | 0.23 |
| 35 | -21.6 ± 7.8 | -65.8 ± 6.7 | 1 | -7.0 ± 3.9 | -115.7 ± 6.0 | 0.34 |
| | | | 2 | -4.4 ± 3.3 | -111.6 ± 7.0 | 0.10 |
| | | | 3 | -14.1 ± 5.6 | -113.8 ± 6.1 | 0.48 |
| | | | 4 | -8.9 ± 5.5 | -108.5 ± 6.4 | 0.08 |
| 36 | -21.4 ± 7.5 | -65.4 ± 6.6 | 1 | -7.3 ± 3.1 | -115.6 ± 6.5 | 0.29 |
| | | | 2 | -7.4 ± 4.9 | -112.2 ± 6.8 | 0.13 |

| | | | | | | |
|---|---|---|---|---|---|---|
| | | | 3 | -7.0 ± 4.2 | -113.2 ± 6.3 | 0.16 |
| | | | 4 | -7.7 ± 3.8 | -117.1 ± 6.5 | 0.43 |
| 38 | -12.2 ± 5.5 | -67.0 ± 6.3 | 1 | -9.2 ± 4.6 | -110.8 ± 6.5 | 0.13 |
| | | | 2 | -3.9 ± 3.4 | -117.3 ± 5.8 | 0.34 |
| | | | 3 | -5.9 ± 3.7 | -111.3 ± 6.6 | 0.10 |
| | | | 4 | -10.3 ± 3.4 | -115.4 ± 6.5 | 0.43 |
| 42 | -21.8 ± 7.7 | -66.1 ± 6.6 | 1 | -9.8 ± 4.6 | -113.6 ± 6.0 | 0.28 |
| | | | 2 | -9.4 ± 3.3 | -110.5 ± 7.0 | 0.13 |
| | | | 3 | -5.0 ± 3.2 | -104.3 ± 6.1 | 0.02 |
| | | | 4 | -7.5 ± 3.7 | -113.4 ± 6.6 | 0.21 |
| | | | 5 | -5.0 ± 3.1 | -117.0 ± 6.1 | 0.37 |
| 50 | -58.1 ± 13.4 | -45.2 ± 7.5 | 1 | -42.6 ± 14.5 | -80.3 ± 7.7 | 0.28 |
| | | | 2 | -41.8 ± 13.1 | -80.6 ± 7.5 | 0.27 |
| | | | 3 | -41.3 ± 10.8 | -81.0 ± 8.2 | 0.29 |
| | | | 4 | -42.6 ± 13.0 | -77.8 ± 9.0 | 0.16 |
| 51 | -54.4 ± 13.0 | -39.9 ± 7.4 | 1 | -44.4 ± 13.2 | -71.2 ± 7.8 | 0.22 |
| | | | 2 | -47.7 ± 11.7 | -72.3 ± 7.8 | 0.40 |
| | | | 3 | -37.4 ± 15.4 | -72.7 ± 8.1 | 0.15 |
| | | | 4 | -34.8 ± 8.3 | -72.9 ± 7.4 | 0.12 |
| | | | 5 | -33.2 ± 13.2 | -73.5 ± 7.1 | 0.11 |
| 52 | -54.7 ± 13.6 | -46.5 ± 7.4 | 1 | -41.9 ± 13.5 | -83.7 ± 8.5 | 0.50 |
| | | | 2 | -35.5 ± 10.0 | -84.2 ± 7.3 | 0.28 |
| | | | 3 | -37.6 ± 12.1 | -78.3 ± 7.9 | 0.09 |
| | | | 4 | -32.4 ± 15.1 | -82.2 ± 8.2 | 0.13 |
| 53 | -54.3 ± 13.0 | -52.8 ± 8.1 | 1 | -36.8 ± 11.4 | -94.2 ± 8.2 | 0.19 |
| | | | 2 | -57.7 ± 14.2 | -88.1 ± 8.1 | 0.41 |
| | | | 3 | -32.2 ± 9.6 | -95.9 ± 7.8 | 0.17 |
| | | | 4 | -23.9 ± 11.4 | -96.2 ± 8.4 | 0.07 |
| | | | 5 | -42.5 ± 11.7 | -90.9 ± 8.7 | 0.16 |
| 54 | -54.0 ± 13.0 | -65.9 ± 8.7 | 1 | -41.7 ± 12.1 | -115.9 ± 8.3 | 0.25 |
| | | | 2 | -27.7 ± 14.6 | -121.1 ± 8.0 | 0.19 |
| | | | 3 | -42.6 ± 12.3 | -112.1 ± 8.9 | 0.11 |
| | | | 4 | -45.1 ± 11.5 | -112.5 ± 11.5 | 0.16 |
| | | | 5 | -36.3 ± 10.5 | -108.9 ± 9.8 | 0.03 |
| | | | 6 | -42.1 ± 12.2 | -116.1 ± 8.7 | 0.27 |
| | | | 7 | -36.5 ± 12.5 | -105.3 ± 8.6 | |
| 55 | -53.3 ± 12.9 | -72.5 ± 8.5 | 1 | -32.7 ± 12.5 | -122.9 ± 9.1 | 0.09 |
| | | | 2 | -38.2 ± 11.6 | -123.4 ± 9.0 | 0.18 |
| | | | 3 | -35.6 ± 10.3 | -124.9 ± 8.8 | 0.20 |
| | | | 4 | -44.1 ± 10.8 | -123.4 ± 9.6 | 0.34 |
| | | | 5 | -28.7 ± 13.4 | -124.2 ± 9.7 | 0.08 |
| | | | 6 | -27.1 ± 16.9 | -126.4 ± 8.6 | 0.11 |
| 56 | -52.6 ± 13.0 | -79.8 ± 9.0 | 1 | -35.3 ± 10.9 | -131.4 ± 9.0 | 0.06 |
| | | | 2 | -33.8 ± 7.8 | -133.8 ± 8.7 | 0.08 |

| | | | | | | |
|---|---|---|---|---|---|---|
| | | | 3 | -35.7 ± 12.2 | -136.4 ± 8.8 | 0.19 |
| | | | 4 | -39.4 ± 11.7 | -139.6 ± 9.4 | 0.60 |
| | | | 5 | -33.1 ± 10.3 | -133.6 ± 9.3 | 0.07 |
| 58 | -57.5 ± 13.6 | -70.5 ± 8.7 | 1 | -48.4 ± 11.4 | -119.7 ± 8.7 | 0.25 |
| | | | 2 | -42.7 ± 14.1 | -120.2 ± 9.4 | 0.15 |
| | | | 3 | -42.5 ± 13.7 | -105.7 ± 9.3 | 0.01 |
| | | | 4 | -38.2 ± 10.7 | -126.1 ± 8.9 | 0.38 |
| | | | 5 | -35.5 ± 11.6 | -124.1 ± 9.1 | 0.18 |
| | | | 6 | -43.5 ± 12.5 | -114.0 ± 10.7 | 0.04 |
| 60 | -46.5 ± 12.8 | -50.9 ± 7.7 | 1 | -41.1 ± 10.7 | -90.1 ± 7.6 | 0.27 |
| | | | 2 | -11.3 ± 9.7 | -95.5 ± 7.4 | 0.04 |
| | | | 3 | -33.8 ± 9.4 | -91.5 ± 7.2 | 0.17 |
| | | | 4 | -35.4 ± 9.4 | -94.1 ± 7.0 | 0.38 |
| | | | 5 | -33.6 ± 10.9 | -90.6 ± 7.8 | 0.14 |
| 61 | -45.7 ± 12.1 | -51.5 ± 7.4 | 1 | -13.6 ± 14.8 | -94.3 ± 7.0 | 0.10 |
| | | | 2 | -25.7 ± 9.0 | -92.2 ± 7.3 | 0.22 |
| | | | 3 | -30.4 ± 9.7 | -89.7 ± 7.3 | 0.20 |
| | | | 4 | -33.2 ± 8.8 | -89.7 ± 7.5 | 0.28 |
| | | | 5 | -32.8 ± 9.9 | -88.5 ± 7.4 | 0.20 |
| 62 | -25.3 ± 8.9 | -50.4 ± 6.4 | 1 | -10.2 ± 7.3 | -85.6 ± 6.3 | 0.15 |
| | | | 2 | -12.9 ± 6.6 | -87.4 ± 6.3 | 0.30 |
| | | | 3 | -13.4 ± 6.8 | -87.7 ± 6.9 | 0.34 |
| | | | 4 | -13.0 ± 6.6 | -86.1 ± 6.4 | 0.22 |
| 64 | -64.0 ± 14.6 | -48.0 ± 8.1 | 1 | -61.7 ± 11.8 | -86.2 ± 8.9 | 0.21 |
| | | | 2 | -59.3 ± 12.5 | -82.4 ± 8.3 | 0.07 |
| | | | 3 | -48.8 ± 12.8 | -88.4 ± 8.7 | 0.09 |
| | | | 4 | -67.7 ± 17.8 | -83.7 ± 9.0 | 0.22 |
| | | | 5 | -70.1 ± 14.4 | -85.5 ± 8.7 | 0.43 |
| 65 | -27.4 ± 9.4 | -62.5 ± 7.0 | 1 | -10.7 ± 4.5 | -108.7 ± 6.0 | 0.16 |
| | | | 2 | -6.9 ± 4.8 | -107.3 ± 6.4 | 0.08 |
| | | | 3 | -11.8 ± 4.8 | -109.5 ± 6.3 | 0.22 |
| | | | 4 | -12.5 ± 4.6 | -108.1 ± 6.2 | 0.17 |
| | | | 5 | -7.9 ± 5.4 | -112.7 ± 6.8 | 0.31 |
| | | | 6 | -9.1 ± 5.5 | -105.9 ± 6.3 | 0.07 |
| 67 | -22.9 ± 8.5 | -69.9 ± 7.0 | 1 | -5.4 ± 4.9 | -123.1 ± 7.7 | 0.16 |
| | | | 2 | -7.4 ± 3.8 | -122.4 ± 6.3 | 0.16 |
| | | | 3 | -10.1 ± 3.9 | -121.2 ± 6.7 | 0.17 |
| | | | 4 | -6.1 ± 5.0 | -123.8 ± 7.5 | 0.21 |
| | | | 5 | -12.9 ± 5.5 | -119.0 ± 7.0 | 0.14 |
| | | | 6 | -10.7 ± 4.6 | -120.2 ± 6.7 | 0.15 |
| 68 | 2.7 ± 1.1 | -68.3 ± 6.2 | 1 | 0.0 ± 0.9 | -118.3 ± 7.9 | 0.52 |
| | | | 2 | 0.9 ± 0.8 | -103.9 ± 8.4 | 0.02 |
| | | | 3 | 0.2 ± 0.9 | -114.7 ± 7.1 | 0.22 |
| | | | 4 | 0.7 ± 0.8 | -105.8 ± 7.0 | 0.03 |

| | | | | | | |
|---|---|---|---|---|---|---|
| | | | 5 | 0.5 ± 0.8 | -112.1 ± 7.1 | 0.12 |
| | | | 6 | 0.4 ± 1.0 | -111.3 ± 9.3 | 0.10 |
| 70 | -68.0 ± 14.7 | -60.2 ± 8.2 | 1 | -35.0 ± 12.6 | -105.8 ± 8.8 | 0.05 |
| | | | 2 | -48.0 ± 16.3 | -106.7 ± 8.4 | 0.23 |
| | | | 3 | -47.3 ± 13.3 | -108.5 ± 8.8 | 0.32 |
| | | | 4 | -46.0 ± 12.7 | -110.0 ± 7.8 | 0.40 |
| 72 | -52.9 ± 12.7 | -47.4 ± 7.7 | 1 | -45.8 ± 13.0 | -84.2 ± 8.6 | 0.27 |
| | | | 2 | -50.3 ± 11.8 | -83.3 ± 8.3 | 0.36 |
| | | | 3 | -37.3 ± 12.7 | -85.1 ± 7.7 | 0.14 |
| | | | 4 | -39.3 ± 10.0 | -86.4 ± 7.1 | 0.23 |
| 73 | -95.2 ± 17.6 | -59.2 ± 10.0 | 1 | -63.6 ± 17.1 | -108.1 ± 10.2 | 0.11 |
| | | | 2 | -55.1 ± 13.2 | -118.8 ± 10.1 | 0.55 |
| | | | 3 | -58.1 ± 16.6 | -113.0 ± 10.5 | 0.20 |
| | | | 4 | -48.5 ± 17.6 | -111.2 ± 9.0 | 0.05 |
| | | | 5 | -37.2 ± 19.1 | -115.4 ± 11.5 | 0.04 |
| | | | 6 | -45.8 ± 22.7 | -113.2 ± 10.6 | 0.06 |
| Test set compounds | | | | | | |
| 9 | -57.0 ± 14.6 | -50.2 ± 8.7 | 1 | -49.1 ± 13.4 | -92.5 ± 9.5 | 0.39 |
| | | | 2 | -40.1 ± 10.1 | -94.3 ± 8.0 | 0.23 |
| | | | 3 | -51.5 ± 11.2 | -87.4 ± 8.9 | 0.15 |
| | | | 4 | -48.7 ± 13.3 | -90.3 ± 9.2 | 0.22 |
| 10 | -65.0 ± 15.4 | -48.9 ± 9.2 | 1 | -45.3 ± 13.2 | -92.2 ± 9.2 | 0.10 |
| | | | 2 | -49.9 ± 13.8 | -90.8 ± 9.0 | 0.12 |
| | | | 3 | -54.2 ± 14.7 | -89.2 ± 9.1 | 0.13 |
| | | | 4 | -56.4 ± 10.1 | -94.4 ± 8.4 | 0.56 |
| | | | 5 | -57.9 ± 13.5 | -86.3 ± 9.7 | 0.10 |
| 11 | -54.7 ± 13.4 | -51.2 ± 7.8 | 1 | -22.5 ± 8.4 | -94.3 ± 6.5 | 0.07 |
| | | | 2 | -40.3 ± 10.7 | -94.2 ± 7.4 | 0.44 |
| | | | 3 | -27.0 ± 10.1 | -92.1 ± 7.3 | 0.06 |
| | | | 4 | -37.2 ± 13.0 | -85.3 ± 8.8 | 0.04 |
| | | | 5 | -47.8 ± 9.6 | -90.4 ± 7.8 | 0.39 |
| 12 | -59.5 ± 15.3 | -50.9 ± 9.0 | 1 | -34.7 ± 14.1 | -98.6 ± 8.2 | 0.13 |
| | | | 2 | -49.0 ± 15.9 | -87.0 ± 9.6 | 0.04 |
| | | | 3 | -34.3 ± 13.3 | -97.3 ± 8.7 | 0.09 |
| | | | 4 | -19.6 ± 15.0 | -100.3 ± 8.0 | 0.04 |
| | | | 5 | -42.6 ± 13.1 | -94.1 ± 9.0 | 0.11 |
| | | | 6 | -41.7 ± 10.9 | -101.8 ± 8.9 | 0.59 |
| 14 | -171.0 ± 34.2 | -78.0 ± 16.3 | 1 | -120.1 ± 24.4 | -161.0 ± 14.5 | 0.04 |
| | | | 2 | -125.2 ± 23.8 | -167.5 ± 13.3 | 0.31 |
| | | | 3 | -71.3 ± 24.3 | -172.9 ± 10.9 | 0.00 |
| | | | 4 | -106.6 ± 32.7 | -163.9 ± 14.3 | 0.02 |
| | | | 5 | -146.2 ± 29.4 | -158.9 ± 15.6 | 0.39 |
| | | | 6 | -154.7 ± 35.2 | -152.9 ± 18.0 | 0.24 |
| 17 | -132.0 ± 29.2 | -77.4 ± 14.1 | 1 | -112.0 ± 18.2 | -152.5 ± 13.9 | 0.53 |

| | | | | | | |
|---|---|---|---|---|---|---|
| | | | 2 | -50.7 ± 18.3 | -168.3 ± 9.6 | 0.03 |
| | | | 3 | -83.3 ± 17.1 | -155.1 ± 11.7 | 0.05 |
| | | | 4 | -100.5 ± 17.8 | -140.5 ± 12.7 | 0.01 |
| | | | 5 | -95.2 ± 17.1 | -158.6 ± 13.4 | 0.38 |
| 20 | -92.7 ± 20.9 | -91.0 ± 11.8 | 1 | -64.3 ± 20.3 | -162.8 ± 12.3 | 0.19 |
| | | | 2 | -56.8 ± 27.7 | -146.9 ± 14.5 | 0.00 |
| | | | 3 | -89.0 ± 17.6 | -151.6 ± 12.4 | 0.20 |
| | | | 4 | -54.8 ± 24.5 | -154.7 ± 17.1 | 0.01 |
| | | | 5 | -52.3 ± 16.1 | -170.2 ± 10.7 | 0.30 |
| | | | 6 | -70.5 ± 15.8 | -161.7 ± 11.5 | 0.29 |
| 29 | -17.1 ± 6.7 | -60.2 ± 6.2 | 1 | -7.7 ± 3.6 | -101.1 ± 5.8 | 0.19 |
| | | | 2 | -7.7 ± 3.8 | -105.0 ± 6.4 | 0.48 |
| | | | 3 | -6.3 ± 3.8 | -102.7 ± 6.1 | 0.23 |
| | | | 4 | -8.1 ± 4.3 | -98.2 ± 6.5 | 0.10 |
| 31 | -21.8 ± 7.7 | -65.8 ± 6.6 | 1 | -6.9 ± 3.4 | -111.5 ± 6.3 | 0.06 |
| | | | 2 | -11.1 ± 3.9 | -116.0 ± 7.1 | 0.27 |
| | | | 3 | -9.2 ± 5.3 | -112.4 ± 6.5 | 0.10 |
| | | | 4 | -8.7 ± 3.8 | -115.6 ± 6.8 | 0.19 |
| | | | 5 | -4.5 ± 3.1 | -116.2 ± 6.2 | 0.14 |
| | | | 6 | -10.6 ± 3.8 | -115.5 ± 6.7 | 0.23 |
| 33 | -30.0 ± 9.7 | -63.6 ± 6.9 | 1 | -12.3 ± 4.0 | -108.8 ± 6.1 | 0.12 |
| | | | 2 | -12.5 ± 6.8 | -109.6 ± 7.1 | 0.14 |
| | | | 3 | -14.5 ± 7.1 | -111.1 ± 7.1 | 0.26 |
| | | | 4 | -19.0 ± 6.6 | -107.8 ± 6.6 | 0.19 |
| | | | 5 | -14.8 ± 7.1 | -109.9 ± 7.2 | 0.20 |
| | | | 6 | -13.2 ± 5.9 | -107.5 ± 6.6 | 0.10 |
| 34 | -21.7 ± 7.8 | -65.1 ± 6.6 | 1 | -5.9 ± 3.7 | -111.4 ± 7.1 | 0.06 |
| | | | 2 | -7.6 ± 4.0 | -116.9 ± 6.5 | 0.27 |
| | | | 3 | -8.1 ± 3.7 | -117.9 ± 7.4 | 0.37 |
| | | | 4 | -6.6 ± 4.4 | -117.7 ± 6.9 | 0.30 |
| 37 | -12.8 ± 6.0 | -66.9 ± 6.2 | 1 | -8.4 ± 3.6 | -117.4 ± 6.9 | 0.46 |
| | | | 2 | -7.4 ± 3.7 | -115.5 ± 6.3 | 0.26 |
| | | | 3 | -4.6 ± 3.8 | -114.4 ± 6.1 | 0.15 |
| | | | 4 | -9.7 ± 4.1 | -111.4 ± 7.0 | 0.13 |
| 39 | -21.3 ± 7.6 | -65.5 ± 6.6 | 1 | -10.3 ± 4.1 | -110.6 ± 7.2 | 0.17 |
| | | | 2 | -8.4 ± 4.1 | -115.9 ± 6.8 | 0.48 |
| | | | 3 | -5.2 ± 3.7 | -114.6 ± 6.6 | 0.25 |
| | | | 4 | -5.8 ± 3.5 | -110.5 ± 6.9 | 0.10 |
| 40 | -21.5 ± 7.6 | -65.8 ± 6.6 | 1 | -11.2 ± 5.7 | -106.1 ± 7.0 | 0.03 |
| | | | 2 | -6.2 ± 3.6 | -119.9 ± 7.1 | 0.49 |
| | | | 3 | -5.0 ± 4.7 | -115.6 ± 6.9 | 0.16 |
| | | | 4 | -7.3 ± 4.1 | -116.7 ± 7.0 | 0.26 |
| | | | 5 | -4.4 ± 2.9 | -112.0 ± 6.5 | 0.06 |
| 41 | -21.7 ± 7.4 | -65.7 ± 6.5 | 1 | -5.5 ± 4.3 | -110.6 ± 6.3 | 0.08 |

| | | | | | | |
|---|---|---|---|---|---|---|
| | | | 2 | -8.3 ± 4.1 | -113.5 ± 7.2 | 0.21 |
| | | | 3 | -5.7 ± 4.3 | -115.2 ± 6.7 | 0.24 |
| | | | 4 | -6.4 ± 3.9 | -117.6 ± 7.1 | 0.45 |
| | | | 5 | -4.3 ± 3.4 | -104.6 ± 6.7 | 0.02 |
| 43 | -21.6 ± 7.6 | -66.4 ± 6.5 | 1 | -5.7 ± 3.6 | -113.6 ± 6.4 | 0.10 |
| | | | 2 | -7.7 ± 4.0 | -117.3 ± 7.4 | 0.30 |
| | | | 3 | -4.6 ± 3.4 | -115.3 ± 6.1 | 0.13 |
| | | | 4 | -4.3 ± 3.7 | -110.1 ± 7.0 | 0.04 |
| | | | 5 | -6.7 ± 3.5 | -119.2 ± 6.4 | 0.42 |
| 57 | -53.7 ± 12.9 | -85.1 ± 9.0 | 1 | -38.4 ± 9.4 | -148.5 ± 9.8 | 0.57 |
| | | | 2 | -29.1 ± 8.9 | -144.9 ± 9.5 | 0.09 |
| | | | 3 | -23.5 ± 13.5 | -145.5 ± 8.4 | 0.06 |
| | | | 4 | -15.8 ± 9.9 | -148.9 ± 8.9 | 0.06 |
| | | | 5 | -37.6 ± 10.9 | -144.9 ± 9.8 | 0.22 |
| | | | 6 | -32.9 ± 9.6 | -132.1 ± 9.9 | 0.01 |
| 59 | -56.1 ± 13.1 | -77.5 ± 8.7 | 1 | -45.8 ± 10.9 | -137.7 ± 9.7 | 0.56 |
| | | | 2 | -30.2 ± 10.2 | -131.6 ± 9.3 | 0.02 |
| | | | 3 | -33.2 ± 12.0 | -128.3 ± 9.0 | 0.02 |
| | | | 4 | -43.3 ± 12.7 | -128.5 ± 9.0 | 0.05 |
| | | | 5 | -42.0 ± 11.4 | -136.1 ± 9.4 | 0.25 |
| | | | 6 | -32.6 ± 11.9 | -136.3 ± 8.5 | 0.10 |
| 63 | -17.6 ± 7.4 | -52.3 ± 6.0 | 1 | -3.1 ± 5.1 | -82.7 ± 8.1 | 0.06 |
| | | | 2 | -2.5 ± 3.8 | -90.0 ± 6.6 | 0.34 |
| | | | 3 | -3.1 ± 2.5 | -89.2 ± 6.0 | 0.29 |
| | | | 4 | -2.7 ± 3.5 | -89.5 ± 5.9 | 0.31 |
| 66 | -20.7 ± 8.3 | -68.2 ± 7.1 | 1 | -3.3 ± 4.0 | -116.8 ± 7.1 | 0.10 |
| | | | 2 | -7.4 ± 4.2 | -117.6 ± 7.2 | 0.19 |
| | | | 3 | -12.9 ± 5.0 | -116.7 ± 7.5 | 0.27 |
| | | | 4 | -6.7 ± 5.6 | -118.6 ± 6.8 | 0.22 |
| | | | 5 | -10.8 ± 4.8 | -116.0 ± 7.6 | 0.18 |
| | | | 6 | -8.0 ± 4.8 | -110.4 ± 6.5 | 0.04 |
| 69 | -16.1 ± 7.6 | -60.6 ± 6.5 | 1 | -4.9 ± 6.0 | -100.8 ± 6.8 | 0.14 |
| | | | 2 | -2.3 ± 2.1 | -102.9 ± 6.0 | 0.17 |
| | | | 3 | -4.6 ± 3.7 | -99.8 ± 6.5 | 0.11 |
| | | | 4 | -3.9 ± 3.0 | -104.0 ± 7.5 | 0.27 |
| | | | 5 | -5.2 ± 3.7 | -103.9 ± 6.6 | 0.31 |
| 71 | -58.0 ± 13.5 | -42.3 ± 7.7 | 1 | -43.4 ± 11.6 | -76.5 ± 7.7 | 0.30 |
| | | | 2 | -31.1 ± 11.6 | -79.8 ± 7.4 | 0.18 |
| | | | 3 | -26.0 ± 12.6 | -80.0 ± 8.1 | 0.11 |
| | | | 4 | -39.4 ± 12.5 | -76.8 ± 9.0 | 0.21 |
| | | | 5 | -38.8 ± 12.5 | -77.0 ± 7.6 | 0.21 |
